# New Fluorescent Probes, Their Spectroscopic Properties, and an Iterative Analysis of Their Complexation with Cyclodextrins

**DOI:** 10.1021/acs.jpcb.5c06255

**Published:** 2026-02-03

**Authors:** Monika Topa-Skwarczyńska, Patryk Szymaszek, Anna Chachaj-Brekiesz, Mariusz Galek, Joanna Ortyl, Roman Popielarz

**Affiliations:** † Cracow University of Technology, 272584 Faculty of Chemical Engineering and Technology, Warszawska 24, 31-155 Kraków, Poland; ‡ Faculty of Chemistry, Jagiellonian University, Gronostajowa 2, 30-387 Kraków, Poland; § Photo HiTech Ltd., Bobrzyńskiego 14, 30-348 Kraków, Poland; ∥ Photo4Chem Ltd., Lea 114, 30-133 Kraków, Poland

## Abstract

Spectroscopic properties of a series of substituted 7-phenylamino-3-(2-pyridyl)­coumarins have been characterized, and their ability to form host–guest inclusion complexes with cyclodextrins has been evaluated by the determination of the corresponding host–guest association constants. It has been found that the pyridylcoumarins form 1:1 host–guest complexes with sulfobutylated β-cyclodextrin (Captisol). Their host–guest association constants vary in the range 17–122 dm^3^ mol^–1^, depending on the type of substituent. The association constant decreases with an increase of the electron-withdrawing character of the substituents, which suggests that the pyridylcoumarins interact with positively charged sites within the cyclodextrin cavity. Moreover, the problem associated with the Benesi–Hildebrand method, commonly used for determination of the host–guest association constants, has been clearly demonstrated and the use of an alternative data workup method, based on consecutive iterations methodology, is presented and explained in detail to enable the application of this methodology also to other experimental data sets, not necessarily related to the Benesi–Hildebrand equation.

## Graphical abstract

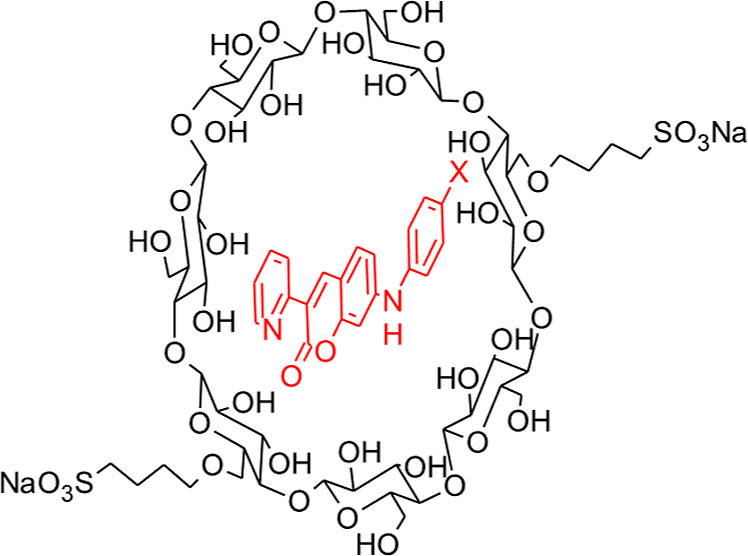

## 1 Introduction

Coumarins are organic compounds that have a range of valuable properties, making them distinctive for a wide range of applications. They were identified as early as at the beginning of the 19th century, and currently more than 1300 different derivatives of their natural origin are known, which are used in many scientific disciplines. Of particular importance is their use in medicine and pharmacy in their broadest sense.^1–6^ It has long been known that coumarin derivatives exhibit anticoagulant,^7–9^ analgesic,^7,10–12^ and even sedative effects.^13,14^ Therefore, drugs based on the coumarin skeleton have appeared on the pharmaceutical market, which are used against varicose veins,^15^ hemorrhoids, ulcers,^16^ ischemic heart disease,^17^ etc. Coumarin rings are also included in drugs against such diseases as Alzheimer’s,^18–20^ Parkinson’s,^21–23^ schizophrenia,^24^ epilepsy,^25^ and depression.^26,27^ On the other hand, there have been many recent scientific reports on their potential use as fluorescent probes or stains for disease diagnosis.^28,29^ However, the often encountered problem with synthetic organic compounds, including coumarin derivatives, is their lack of stability or change of their properties under the influence of various environmental factors. This problem has been partially eliminated by the formation of host–guest inclusion complexes, where cyclodextrins can play the role of hosts. Although their discovery took place more than 100 years ago, cyclodextrins have now become the subject of numerous studies. Cyclodextrins, which are cyclic oligosaccharides composed of six or more glucose units, have garnered significant interest in the scientific community due to their ability to form inclusion complexes with a variety of guest molecules. The structure of cyclodextrins, resembling a conical shape, allows encapsulation of hydrophobic guest molecules within the hydrophobic cavity, while leaving hydrophilic hydroxyl groups on the outside, which makes these complexes water-soluble.^30,31^

The formation of inclusion complexes with cyclodextrins is a phenomenon of great importance in the fields of pharmacy, analytical chemistry, and materials engineering. In pharmacy, cyclodextrins can enhance bioavailability of active substances, protect them from enzymatic degradation, and reduce their toxicity.^32^ In materials engineering, the ability of cyclodextrins to modify the surface properties of materials finds applications in the design of modern drug delivery systems and adsorptive materials.^33^ However, the application of cyclodextrins is much broader. Inclusion complexes with cyclodextrins are studied for their ability to stabilize chemical compounds and improve their solubility in water, which is crucial in food and cosmetic industries.^34^ Numerous cases have been described in the literature, where cyclodextrins play a role of carriers for active substances, catalysts for chemical reactions, and components of controlled release systems.^35,36^

One of the challenges associated with cyclodextrins is the precise determination of the strength of their interactions with guest molecules, particularly those of a fluorescent nature. The strength of the interaction is crucial because the binding strength in inclusion complexes directly influences their stability and functional efficiency. Various techniques, such as UV/vis absorption spectroscopy, fluorescence emission spectroscopy, fluorescence microscopy, NMR spectroscopies, and chromatographic methods, have been applied for the assessment of cyclodextrin interactions with various molecules, enabling determination of association constants and binding enthalpies.^37–39^ In the studies of interactions of cyclodextrins with fluorescent molecules, special attention is given to analytical techniques that measure changes in fluorescence intensity in the presence of cyclodextrins. Understanding the mechanisms of cyclodextrin interactions with guest molecules within host–guest inclusion complexes and the precise determination of host–guest association constants are essential for further advancement of the applications of these interesting compounds.

In this article, spectroscopic properties of a series of new 7-phenylamino-3-(2-pyridyl)­coumarins, containing various electron-donating or electron-withdrawing substituents, for the role of fluorescent molecular sensors are described, and the effect of substituents on those properties is evaluated. Moreover, the results of our studies on the interaction of these fluorophores with a commercial β-cyclodextrin (β-CD) (Captisol) in an attempt to generate host–guest inclusion complexes are reported. Finally, a problem associated with the direct application of the commonly used Benesi–Hildebrand method for the determination of host–guest association constants from experimental data, covering more than 1 order of magnitude of host concentrations, is demonstrated, and an alternative approach to the data workup that affords much more reliable host–guest association constants is described in detail.

## 2 Materials and Methods

### 2.1 Synthesis of the Compounds Studied

A series of new 7-phenylamino-3-(2-pyridyl)coumarin derivatives (PAPC) were synthesized and studied. Their structures, chemical names, and assigned acronyms are collected in Table 1. All of the PAPCs were synthesized from commercially available reagents, as depicted in Scheme 1. The reagents were purchased from Fluorochem, POCh, Sigma-Aldrich, Chempur, or Combi-Blocks. Solvents used for spectroscopic studies were of spectroscopic grade and were purchased from Sigma-Aldrich.

**Table 1:**
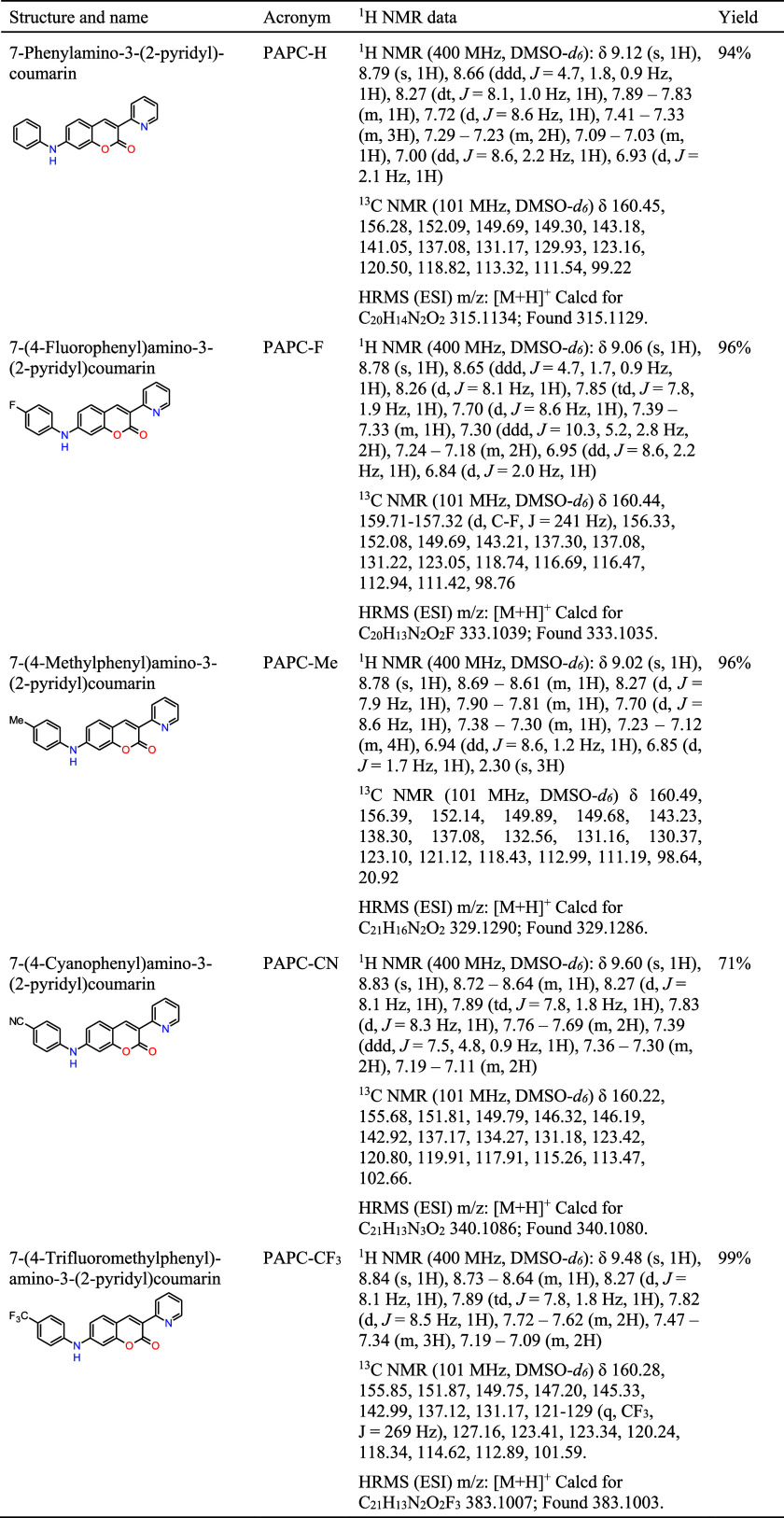
3-(2-Pyridyl)­coumarins Studied

**Scheme 1:**
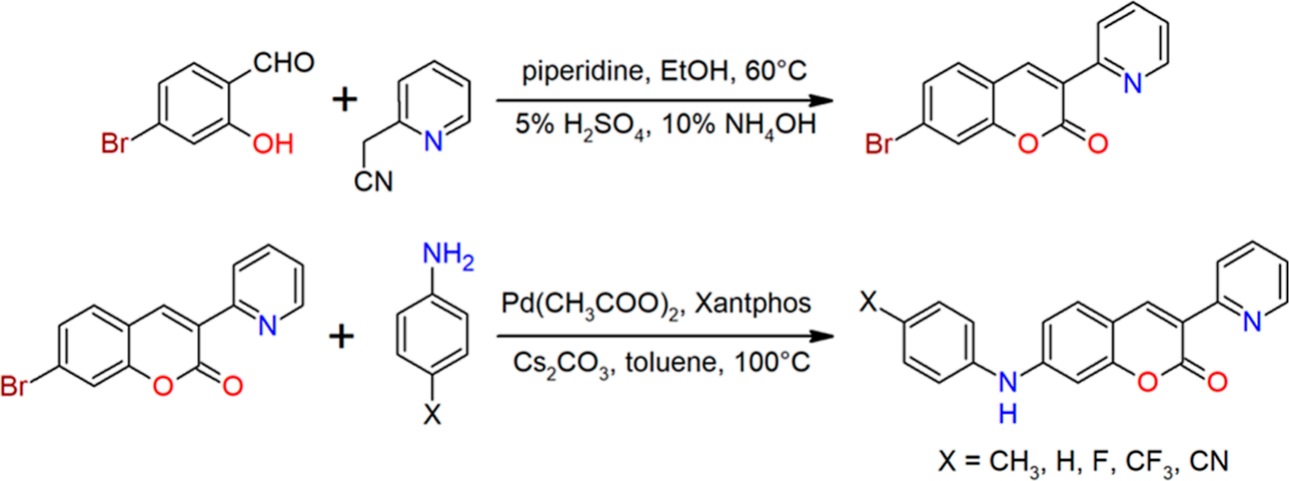
Synthesis of the Derivatives Studied

The syntheses of the derivatives of 3-(2-pyridyl)­coumarin were carried out in two steps. First, 7 bromo-3-(2-pyridyl)­coumarin was synthesized. For this purpose, 4-bromo-2-hydroxybenzaldehyde (2.50 g, 12.5 mmol) and (2-pyridyl)­acetonitrile (1.48 g, 12.5 mmol) were dissolved in a minimum amount of ethanol (20 mL). The solution was heated to 60 °C, and 0.1 mL of piperidine was added. The mixture was stirred overnight at room temperature. Then, 7 mL of 5% H_2_SO_4_ was added and the mixture was refluxed for 5 h. After being cooled to room temperature, the reaction mixture was neutralized with 10% aqueous ammonia. The resulting precipitate was filtered off and recrystallized from ethanol.

The bromo-derivative obtained in the first step (151 mg, 0.50 mmol) was placed in a pressurized vial, and 8 mg of palladium acetate, 24 mg of Xantphos ligand, 244 mg of cesium carbonate, and 6 × 10^–4^ mol of an aniline derivative were added. Depending on the desired target product, the following aniline derivatives were applied: aniline (56 mg), 4-fluoroaniline (67 mg), p-toluidine (64 mg), 4-aminobenzonitrile (71 mg), or 4-(trifluoromethyl)­aniline (97 mg). Then, 3 mL of toluene was added. The mixture was purged with nitrogen and stirred at 100 °C overnight. Next, the reaction mixture was evaporated to dryness and the residue was adsorbed on silica gel. The product was purified on a chromatography column using chloroform as the eluent.

Structure and purity of synthesized products were confirmed by ^1^H NMR, ^13^C NMR, and HR-MS spectroscopies (Table 1 and Figures S1–S11 in the Supporting Information). The NMR spectra were recorded in DMSO-*d*_6_ on an Avance III HD (Bruker) NMR spectrometer at 400 and 101 MHz for ^1^H NMR and ^13^C NMR spectra, respectively. Chemical shifts were referenced to the signal from residual solvent protons and are reported in parts per million relative to the TMS standard. High-resolution mass spectra were recorded on a Shimadzu LabSolutions LC–MS system.

### 2.2 Measurements of Absorption Spectra

UV–vis absorption spectra of the compounds studied were recorded by using a fiber optics spectrometer (Silver Nova TEC, from StellarNet Inc., USA) and a broadband deuterium-halogen lamp (model L10671, from Hamamatsu Photonics). Measured solutions were placed in a quartz cuvette with a 1 cm optical path and inserted into an appropriate cuvette holder. The light from the light source entering the input port of the cuvette holder and the light exiting the cuvette via the holder output port were transmitted by a pair of quartz fiber optic cables with a 1 mm core (F1000-UVVis-SR-1, from StellarNet, Inc.). As this was a single beam measurement system, the fiber optic cables were not moved during measurements of the tested solutions and pure solvent. The spectrometer was controlled by a microcomputer, where the measured spectra were stored directly in a spreadsheet program (Excel), using appropriate macros. The absorption spectra were recorded in acetonitrile at 21 °C. The concentration of the compounds studied in the solutions used for the absorption spectra measurements was in the range 5.2 × 10^–5^ to 6.6 × 10^–5^ mol dm^–3^, which provided a maximum absorbance magnitude of the order of 1.0, which was in the middle of the spectrometer dynamic range.

### 2.3 Measurements of Fluorescence Spectra

Fluorescence emission spectra of the compounds studied were recorded in acetonitrile for identical concentrations of 1.0 × 10^–5^ mol dm^–3^, using a Quanta Master 40 spectrofluorimeter, at a right angle configuration. To obtain emission spectra, all of the analyzed compounds were excited with the same wavelength of 390 nm, which approximately corresponded to the absorption maximum of the long-wavelength band of the fluorophores studied.

### 2.4 Measurements of Fluorescence Intensity to Determine Association Constants of the Fluorophores with Cyclodextrins

A Tecan Infinite 200 PRO NanoQuant multilevel microplate reader was applied for the measurement of fluorescence intensity from the solutions containing a constant concentration of a fluorophore and variable concentrations of cyclodextrins. This reader was capable of the measurement of fluorescence intensity from the samples contained within particular wells of a 96-well microplate, in a front-face configuration. It was equipped with a microplate shaker with controllable shaking speed and direction that enabled the preparation of the sample solutions directly within the plate wells by micropipeting and mixing the components before fluorescence measurement. Moreover, the reader was equipped with a temperature controller to keep the temperature constant during the measurements, so all measurements were done at 25 °C.

### 2.5 Preparation of the Samples for Testing

First, working solutions of the fluorophores, containing about 5 mg of fluorophore per 250 mL of distilled water, were prepared in volumetric flasks, using an ultrasound bath to speed up dissolution of the fluorophores in water. Exact concentrations of the fluorophores in the fluorophore working solutions were in the range of 2.6 × 10^–5^ to 3.2 × 10^–5^ mol dm^–3^. In a similar way, 100 mL of working solutions of cyclodextrins in distilled water was prepared, but three different working solutions with concentrations that varied by an order of magnitude were prepared from each cyclodextrin to cover the broadest possible range of cyclodextrin concentrations in the samples, from 0 to the limit of cyclodextrins’ solubility in water. For example, concentrations of Captisol in the Captisol working solutions were 4.96 × 10^–2^, 4.96 × 10^–3^, and 4.96 × 10^–4^ mol dm^–3^, respectively. Next, 0.125 mL of a fluorophore working solution was added to a series of wells on a 96-well plate, followed by addition of decreasing volumes of the cyclodextrin working solutions (i.e., 0.15, 0.125, 0.10, 0.080, 0.060, 0.040, 0.020, and 0.010 mL) and complementary volumes of pure water (i.e., 0, 0.025, 0.050, 0.070, 0.090, 0.11, 0.13, and 0.14 mL), so that the total volume of the sample in each well was the same (0.275 mL). Each sample was prepared in triplicate to increase the measurement accuracy and eliminate possible coarse errors that might have resulted from a possible imprecise movement of the reading head to the next well position. Moreover, reference solutions containing the same constant concentration of the fluorophore, but no cyclodextrin, were measured in 6 wells. In this way, 78 samples with constant fluorophore concentrations and varied cyclodextrin concentrations were tested sequentially on the microplate reader for each fluorophore/cyclodextrin pair (i.e., 8 different volumes of cyclodextrin working solution per each of 3 cyclodextrin working solutions of different concentrations, each sample in triplicate, plus 6 blanks with no cyclodextrin).

## 3 Results and Discussion

### 3.1 Spectroscopic Properties of 7-Phenylamino-3-(2-pyridyl)­coumarins

The fluorophores reported in this work were designed for their potential application as fluorescent probes or stains for biochemical or medical purposes. For such applications, it is extremely important to know their spectral characteristics. Hence first, we measured their absorption and fluorescence spectra. From the spectra we determined characteristic spectral parameters, such as the positions of absorption maxima, the wavelength of maximum fluorescence intensity, extinction coefficient (ε_max_) of long-wavelength absorption band, Stokes shift, and the energy gap between lowest vibrational level of their excited singlet state and lowest vibrational level of the corresponding ground state (*E* ^00^ energy).

Figures 1 and 2 show the absorption and fluorescence spectra of the compounds studied, respectively, while characteristic spectral parameters are collected in Table 2. It can be observed that all of the 7-phenylamino-3-(2-pyridyl)­coumarins absorb light in the UV range with a significant absorption tail reaching up to 470 nm in the visible light range. This is very important for biomedical applications of fluorophores because the possibility of excitation of fluorescent probes in the visible light range makes them applicable for use in living organisms, where irradiation with UV light is harmful.

**Figure 1:**
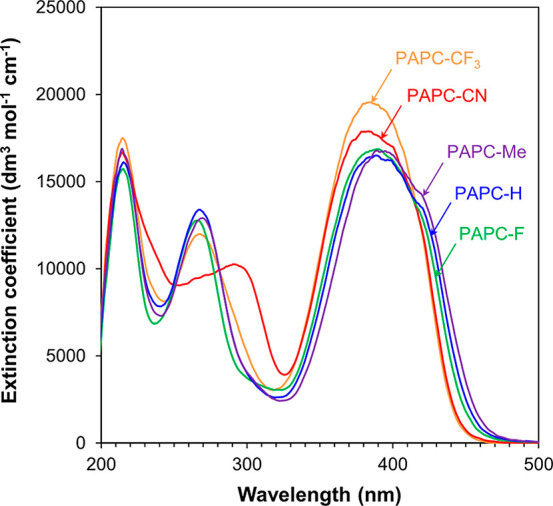
Absorption spectra of the fluorophores studied in acetonitrile.

**Figure 2:**
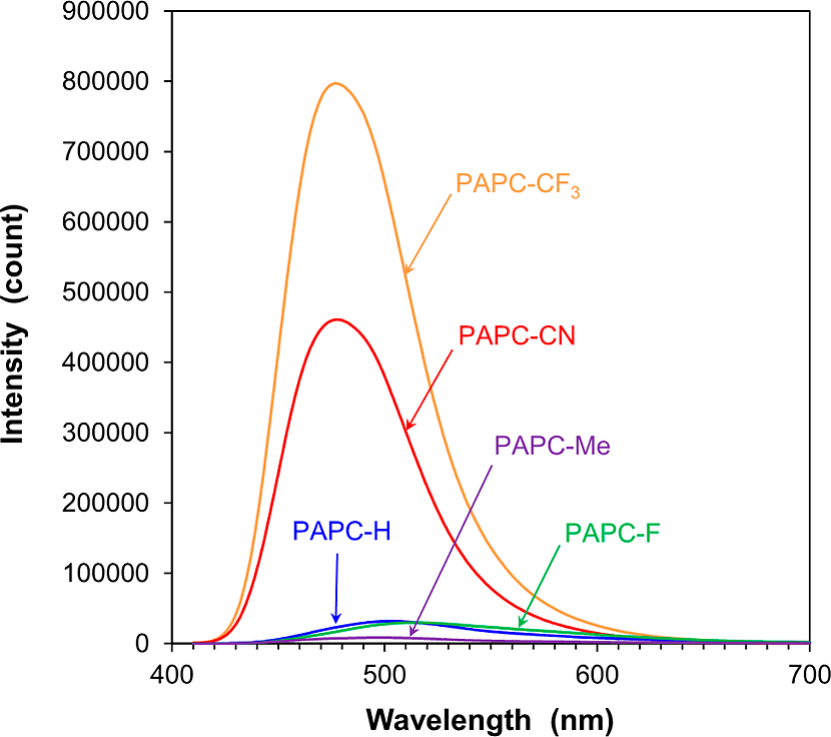
Comparison of fluorescence spectra of the fluorophores studied in acetonitrile, at the same concentration (1.0 × 10^–5^ mol dm^–3^) and excitation wavelength (390 nm).

**Table 2:** Characteristic Spectral Parameters of the 7-Phenylamino-3-(2-pyridyl)­coumarins

compound	λ_max‑ab_ ^a^ (nm)	ε_max_ ^b^ (dm^3^ mol^–1^ cm^–1^)	λ_max‑em_ ^c^ (nm)	*I*_max_ ^d^ (count)	Φ_rel_ ^e^	Stokes shift (cm^–1^)	*E* ^00^ ^f^(kJ mol^–1^)
PAPC-Me	214, 270, 395	16,800	497	8380	0.27	5200	268
PAPC-H	215, 268, 389	16,500	503	31,800	1.00	5800	267
PAPC-F	215, 266, 390	16,900	513	29,800	1.09	6100	267
PAPC-CF_3_	215, 267, 385	19,600	477	797,000	18.1	5000	274
PAPC-CN	214, 291, 383	17,900	476	461,000	10.5	5100	273

a Wavelengths of all absorption maxima.

b Molar extinction coefficient for the long-wavelength absorption band.

c Wavelength of the maximum emission intensity.

d Maximum fluorescence intensity of 1.0 × 10^–5^ mol dm^–3^ solution at 390 nm excitation wavelength, in relative units.

e Fluorescence quantum yield relative to PAPC-H, determined from the emission peak areas.

f Excited singlet state energy relative to the ground state (experimental HOMO–LUMO bandgap).

Long-wavelength absorption maxima of the 7-phenylamino-3-(2-pyridyl)­coumarins fall in the near-UV range, close to 390 nm. The attachment of substituents at the para-position of the phenylamine ring only slightly affects the absorption characteristics, and a slight shift of the absorption spectra toward shorter wavelengths is observed for the derivatives having electron-accepting substituents (i.e., PAPC-CN and PAPC-CF_3_), while electron-donating substituents, such as the methyl group in PAPC-Me, shift the absorption spectrum in the opposite direction. On the other hand, the strong negative inductive effect of the fluorine substituent in PAPC-F is compensated by the strong positive mesomeric effect of the nonbonding electrons on the fluorine atom, as indicated by the magnitude of its Hammett substituent constant σ_p_ (i.e., σ_p(F)_ = 0.06, which is very close to that of the hydrogen substituent: σ_p(H)_ = 0, Table 4). Consequently, the position of the long-wavelength absorption maximum of PAPC-F is also very close to that of PAPC-H (Table 2). Moreover, the data in Table 2 indicate that the substituents do not significantly affect the magnitude of the extinction coefficient of the long-wavelength absorption band, probably because that band comes mostly from electronic transitions within the coumarin moiety, which is separated from the phenyl ring, where the substituents are located, by an NH group that prevents direct conjugation. Consequently, the largest extinction coefficient (i.e., that of PAPC-CF_3_) is greater than the lowest coefficient (i.e., that of PAPC-H) by less than 20%.

**Table 3:** Example Data for PAPC-CF_3_ at a Constant Fluorophore Concentration: [*F*]_o_ = 1.190 × 10^–5^ mol dm^–3^

No.	[CD]_o_ (mol dm^–3^)	*I* ^a^ (count)	1/[CD]_o_ (mol^–1^ dm^3^)	1/(*I* – *I*_o_) (count^–1^)	*I*calc-BH^b^ (count)	*I*calc-exact^c^ (count)
1.	2.71 × 10^–2^	39,368	37.0	2.878 × 10^–5^	17,085	17,085
2.	2.25 × 10^–2^	34,202	44.4	3.380 × 10^–5^	16,858	16,857
3.	1.80 × 10^–2^	30,847	55.4	3.813 × 10^–5^	16,532	16,531
4.	1.44 × 10^–2^	25,689	69.3	4.746 × 10^–5^	16,149	16,148
5.	1.08 × 10^–2^	21,979	92.4	5.760 × 10^–5^	15,562	15,560
6.	7.21 × 10^–3^	17,086	139	8.021 × 10^–5^	14,550	14,547
7.	3.61 × 10^–3^	12,539	277	1.262 × 10^–4^	12,394	12,388
8.	1.80 × 10^–3^	10,103	554	1.823 × 10^–4^	10,040	10,031
9.	2.71 × 10^–3^	11,227	370	1.513 × 10^–4^	11,411	11,403
10.	2.25 × 10^–3^	10,227	444	1.783 × 10^–4^	10,787	10,779
11.	1.80 × 10^–3^	9219	554	2.173 × 10^–4^	10,040	10,031
12.	1.44 × 10^–3^	8468	693	2.597 × 10^–4^	9327	9318
13.	1.08 × 10^–3^	8483	924	2.587 × 10^–4^	8481	8472
14.	7.21 × 10^–4^	6679	1386	4.852 × 10^–4^	7459	7452
15.	3.61 × 10^–4^	6030	2772	7.082 × 10^–4^	6202	6197
16.	1.80 × 10^–4^	5719	5544	9.082 × 10^–4^	5459	5455
17.	2.71 × 10^–4^	5725	3696	9.031 × 10^–4^	5842	5837
18.	2.25 × 10^–4^	5646	4436	9.723 × 10^–4^	5653	5649
19.	1.80 × 10^–4^	5530	5544	1.097 × 10^–3^	5459	5455
20.	1.44 × 10^–4^	5276	6930	1.521 × 10^–3^	5299	5296
21.	1.08 × 10^–4^	5068	9241	2.221 × 10^–3^	5135	5133
22.	7.21 × 10^–5^	4983	13,861	2.740 × 10^–3^	4967	4966
23.	3.61 × 10^–5^	4705	27,722	1.147 × 10^–2^	4795	4794
24.	1.80 × 10^–5^	3027	55,444	–6.286 × 10^–4^	4707	4706
25.	0	4618	-	-	-	4618

a Real fluorescence intensity measured experimentally.

b The fluorescence intensity calculated on the basis of eq 3, using the constants determined by the Benesi–Hildebrand method.

c The fluorescence intensity calculated on the basis of the exact relationship between fluorescence intensity and cyclodextrin concentration (eq 12), using the constants determined by the Benesi–Hildebrand method.

**Table 4:**
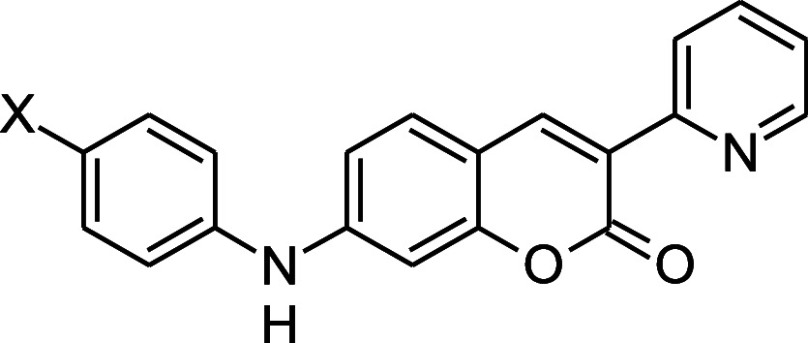
Comparison of the Pyridylcoumarin/Cyclodextrin Association Constants (in dm^3^ mol^–1^), Determined by the Limited Benesi–Hildebrand Method (*K*_BH‑ltd_) and the Consecutive Iterations Method (*K*_CI_), with the Hammett Substituent Constants (σ_p_)

compound	*K*_BH‑ltd_	*K*_CI_	X	σ_p_
PAPC-Me	54 ± 18	122 ± 25	Me	–0.17
PAPC-H	47 ± 11	45 ± 4	H	0
PAPC-F	40 ± 6	60 ± 7	F	0.06
PAPC-CF_3_	22 ± 4	31 ± 5	CF_3_	0.54
PAPC-CN	16 ± 2	17 ± 2	CN	0.66

The effect of substituents on the position of emission spectra of the compounds studied is more pronounced than that on the corresponding absorption spectra and more complex. Strong electron-withdrawing substituents, such as CF_3_ and CN, that shifted absorption spectra toward shorter wavelengths also shifted the corresponding emission spectra in the same direction. However, the weak electron-withdrawing fluorine substituent that affected little the position of the absorption spectrum of PAPC-F compared to PAPC-H shifted the emission spectrum of PAPC-F toward longer wavelengths. This suggests that in the PAPC-F excited state, the positive mesomeric effect of the nonbonding electrons on the fluorine substituent is much stronger than the negative inductive effect of the fluorine atom, causing extensive delocalization of the nonbonding electrons that significantly lowers the energy of the excited state. Moreover, it is interesting to note that the methyl substituent shifted the fluorescence spectrum of PAPC-Me toward shorter wavelengths compared to that of PAPC-H (Table 2). This suggests that simple substituent effect rules that are applicable to absorption spectra are not necessarily applicable to the corresponding fluorescence spectra because the excited states are more affected by substituents, but in a less predictable way.

Fluorescence intensity, measured for the same fluorophore concentration under identical measurement conditions is more than an order of magnitude higher for the fluorophores substituted with electron-withdrawing substituents, such as CF_3_ and CN, than that of the nonsubstituted molecule (PAPC-H). Substitution with a fluorine atom (as in PAPC-F) did not affect the fluorescence intensity compared to the nonsubstituted compound (PAPC-H), while the substitution with a methyl group (as in PAPC-Me) decreased the fluorescence intensity further, four times compared to PAPC-H. The strong enhancement of fluorescence intensity in the derivatives containing electron-withdrawing substituents can be attributed to the push–pull effect between the electron-withdrawing substituents and the secondary amine group in the phenylamino moiety of 7-phenylamino-3-(2-pyridyl)­coumarins in the excited state, which stabilizes the excited state, making it less prone to excitation energy dissipation into radiationless processes. In the case of nonsubstituted or methyl-substituted molecules, the push–pull effect is not possible, while the fluorine substituent in PAPC-F is a very weak electron-acceptor (i.e., its Hammett σ_p_ constant is only 0.06), too weak to be effective in the push–pull interaction. However, at the moment, the attribution of the observed effect of substituents on fluorescence intensity to the “push–pull” effect is only a hypothesis that will need verification in the future, by a detailed study of intramolecular charge transfer processes within the PAPC excited states, and possibly also by the excited states modeling. Regarding the four times lower fluorescence intensity of PAPC-Me compared to PAPC-H, it results from the known ability of methyl groups to dissipate excitation energy of fluorophores into vibrational energy levels of three C–H bonds present in that group, which lowers the fluorescence quantum yield of the fluorophores.

Relative fluorescence quantum yields of the fluorophores studied follow the same trend as the fluorescence intensities (Table 2) because the fluorescence intensities measured at the emission peak maximum for identical fluorophore concentrations and the same excitation conditions are proportional to the fluorescence quantum yields. The large difference in the fluorescence quantum yields of the compounds studied indicates that from a practical point of view only PAPC-CN and PAPC-CF_3_ may be suitable for application as fluorescent probes or stains because the excited states of the other fluorophores (i.e., PAPC-Me, PAPC-H, and PAPC-F) are mostly quenched by radiationless transition processes to the ground state and little fluorescence is emitted. At least 10-times higher excitation light intensity would be required for the weakly emitting probes to achieve the same emission intensity as that of PAPC-CN or PAPC-CF_3_, which could be harmful to biological tissues.

The magnitude of the Stokes shift of 7-phenylamino-3-(2-pyridyl)­coumarins is typical for organic fluorophores and is affected little by the substituents (Table 2) because the substituent effect on absorption spectra acts in the same direction as that on emission spectra, so the difference between emission and absorption peak maxima is affected little. Also the experimental HOMO–LUMO bandgap energy (*E* ^00^, Table 2), calculated from the wavelength of intersection of the normalized absorption and fluorescence spectra, is not influenced significantly by the substituents. However, it can be noticed that electron-withdrawing substituents, such as CN and CF_3_ groups, slightly increase the *E* ^00^ energy (i.e., by several kJ/mol), compared to that of the nonsubstituted derivative PAPC-H (Table 2), because usually electron-withdrawing substituents have a tendency to raise the excited state energy more than the corresponding energy of the ground state.

In comparison to other 7-substituted-2-pyridylcoumarins reported in literature,^40,41^ the main advantage of the 7-phenylamino-3-(2-pyridyl)­coumarins is their extended light absorption range reaching visible light, which is important for their potential biochemical applications.

### 3.2 Determination of Association Constants of the Fluorophores Studied with Captisol

Complexation of the compounds studied with the following commercially available cyclodextrins was attempted: α-cyclodextrin (α-CD), nonmodified β-cyclodextrin (β-CD), γ-cyclodextrin (γ-CD) and sulfobutylated-β-cyclodextrin (SBE-β-CD, called Captisol). α-CD, β-CD, and γ-CD were purchased from Cyclolab, while Captisol was obtained from LIGAND Pharmaceuticals. However, it turned out that only SBE-β-CD (Captisol) provided reliable association constants for all of the fluorophores studied. Therefore, the quantitative data reported in this article concern only the complexation of the compounds studied with the modified β-CD, called Captisol.

The addition of α-CD to an aqueous solution containing the pyridylcoumarins studied did not cause a significant increase of the fluorescence intensity (i.e., at the highest concentration of α-CD, the intensity increased by less than 10% compared to the intensity in the absence of α-CD). This indicates that α-CD practically does not form inclusion complexes with the fluorophores studied, probably due to the too small size of the α-CD cavity compared to the size of the pyridylcoumarin molecules.

In the case of nonmodified β-CD, the fluorescence intensity increase was significantly higher than that in the presence of α-CD, which indicated that the β-CD formed inclusion complexes with the pyridylcoumarins, but the intensity increase was not high enough for precise determination of the corresponding association constants by the fluorescence-based method applied in this study because the experimental errors associated with measurements of small intensity differences were comparable to the magnitude of the differences measured. This was caused mainly by the low solubility of the β-CD in water, which was the lowest among the cyclodextrins studied. When the cyclodextrin solubility and its association constant with a fluorophore are low, the conversion of free fluorophore molecules to its complex with cyclodextrin at the cyclodextrin concentration close to saturation is also low. Then the fluorescence intensity enhancement even at the highest achievable cyclodextrin concentration is small, which makes the measurement of the small intensity differences difficult and prone to experimental errors.

Captisol is a modified β-CD, which is a sulfobutyl ether of β-CD, containing on average two sulfobutyl groups in the form of sodium salts per β-CD moiety. Introduction of the ionic substituents into β-CD structure made the Captisol about 40 times more soluble in water compared to nonmodified β-CD. Much better solubility of Captisol allowed us to achieve its much higher concentrations in water that significantly increased the equilibrium concentration of the Captisol/pyridylcoumarin complexes and consequently increased measured fluorescence intensity differences, which enabled reliable determination of the corresponding association constants.

We also observed significant enhancements of fluorescence intensity in the presence of γ-CD, which were comparable in magnitude to those observed in the case of Captisol. However, in the case of γ-CD, the relationships between fluorescence intensity and γ-CD concentration curved upward with the increase of the γ-CD concentration. The data obtained in the presence of γ-CD did not fit to the Benesi–Hildebrand equation for 1:1 host–guest complexes and did not match well the corresponding equation for 2:1 host–guest complexes at high γ-CD concentrations. This suggests that γ-CD may form mixtures of 1:1 and 2:1 host/guest complexes with the pyridylcoumarins at various proportions, depending on the γ-CD concentration, but the γ-CD interaction with the pyridylcoumarins is not strong enough to form exclusively 2:1 host/guest complexes, even at the highest achievable γ-CD concentrations. It is well-known that γ-CD has a larger cavity size than β-CDs. Hence, it is possible that γ-CD is capable of forming to some extent 2:1 host/guest complexes with one γ-CD molecule incorporating the phenylamino moiety of the 7-phenylamino-3-(2-pyridyl)­coumarins, while another γ-CD molecule is able to contain the remaining 3-(2-pyridyl)­coumarin moiety.

The association constant (*K*) of host–guest inclusion complexes is the basic parameter characterizing the strength of interaction of the host molecules with the guest ones. In the case of interactions of cyclodextrins with nonionic organic molecules, usually 1:1 host–guest complexes are formed because in most cases no more than one organic guest molecule fits within the cyclodextrin cavity, in particular when the strengths of interaction between the cyclodextrin and the guest molecule is not very high. Only in exceptional cases, where the association constants are very large, there is a possibility of formation of complexes with a higher than 1:1 stoichiometry. Therefore, for the purpose of this study, initially it was assumed that the compounds studied formed only 1:1 complexes with Captisol because at the association constant magnitudes found in this study, the probability of formation of complexes with other stoichiometry was very low.

For 1:1 cyclodextrin–fluorophore complexes the association constant (*K*) is defined by eq 1

1 $$K=\frac{\left[FCD\right]}{\left[CD\right]\left[F\right]}$$

where: [F], [CD], and [FCD] represent molar concentrations of fluorophore, cyclodextrin, and the corresponding cyclodextrin–fluorophore host–guest complex at equilibrium, respectively.

Cyclodextrins do not absorb light within the visible range and do not fluoresce, while the 7-phenyl-3-(2-pyridyl)­coumarins turned out to be fluorescent. Moreover, it was observed that the fluorescence intensity increased upon addition of the pyridylcoumarins solutions in water, except for α-CD. This indicated that the pyridylcoumarins interacted with most of the cyclodextrins to form inclusion complexes. Therefore, the method based on measurements of fluorescence intensity was selected for the determination of the association constants of cyclodextrins with the compounds studied.

Theoretically, either the concentration of the fluorophore (guest) could be kept constant, while varying the concentration of cyclodextrin (host), or vice versa, the concentration of cyclodextrin could be set constant, while varying the concentration of the other component. However, fluorescence intensity is approximately linearly proportional to a fluorophore concentration only for very low concentrations, where the magnitude of excitation light absorption by the fluorophore is also very low, so that the measured sample is illuminated as uniformly as possible without significant gradients of excitation light intensity along the sample thickness. Therefore, it is better to keep the fluorophore concentration low and constant while changing the cyclodextrin concentration to avoid significant changes of excitation light intensity within the sample measured.

The enhancement of the fluorescence intensity of fluorophores upon complexation with cyclodextrins can be attributed to the following two factors:

1. Incorporation of a fluorophore molecule inside a cyclodextrin cavity restricts free motions of fluorophore segments, which are responsible for dissipation of excitation energy via nonradiative decay mechanisms. Consequently, upon immobilization of a fluorophore within an inclusion complex, fluorescence intensity increases because of the increase of the overall quantum yield of fluorescence due to the inhibition of the nonradiative excited states decay processes.
2. Fluorescence quantum yield of most of nonionic fluorophores in aqueous solutions is usually lower than that in nonpolar solvents. Hence, when a fluorophore molecule is incorporated inside a cyclodextrin cavity, it becomes surrounded by a much less polar environment, caused by the cyclodextrin structure, than the fluorophore molecules freely floating within the aqueous solution. Consequently, the fluorescence quantum yield of cyclodextrin-fluorophore complexes is higher than that of noncomplexed fluorophore molecules and the overall fluorescence intensity increases upon increase of cyclodextrin concentration.

#### 3.2.1 Application of the Benesi–Hildebrand Method

The Benesi–Hildebrand method is the method used most often for the determination of association constants of cyclodextrins with guest molecules, particularly in systems with low to moderate binding strength. It was originally developed for the determination of association constants between various molecules based on changes in absorbance data of their solutions, measured by UV–vis absorption spectroscopy.^42^ Later on, it was modified for the interpretation of the data based on fluorescence measurements. In fact, the Benesi–Hildebrand method has become particularly valued for its simplicity and ease of interpretation, making it a commonly used approach in studies of interactions of cyclodextrin with various molecules. However, we have noticed that even though the Benesi–Hildebrand method seems to be simple, it may lead to different values of the host–guest association constants (*K*) for the same host–guest systems, if experimental data are not treated with caution.

In order to demonstrate the problem, example data, obtained for PAPC-CF_3_, are collected in Table 3 and their interpretation by the Benesi–Hildebrand method is discussed. The experimental data obtained for the other pyridylcoumarins are included in the Supporting Information.

The core equation of the Benesi–Hildebrand method, modified to fit to fluorescence measurements instead of absorbance measurements, is represented by eq 2.^43^

2 $$\frac{1}{I-I_{o}}=\frac{1}{\left(I_{1}-I_{o}\right)K}\cdot \frac{1}{{\left[CD\right]}_{o}}+\frac{1}{I_{1}-I_{o}}$$

where: [CD]_o_—total concentration of the cyclodextrin in solution (i.e., both in free and complexed form), *I*_o_ and *I*—fluorescence intensity of the fluorophore in the absence and in the presence of various concentrations of cyclodextrin, respectively, *I*_1_—maximum fluorescence intensity that would be observed, if all fluorophore molecules were complexed with cyclodextrin, and *K*—cyclodextrin/fluorophore association constant.

Hence, by plotting the 1/(*I* – *I*_o_) versus reciprocal concentration of cyclodextrin (1/[CD]_o_), a straight line should be obtained for a 1:1 fluorophore/cyclodextrin complex. Then, the association constant (*K*) may be calculated by the division of the line intercept by the line slope, while the maximum fluorescence intensity (*I*_1_) is calculated from the intercept using linear regression to fit the line to experimental data points.

Figure 3 shows the Benesi–Hildebrand plot for all data points included in Table 3, except for data 23 and 24. The data points 23 and 24 were not taken into account because the data 24 provided a negative value of *I*–*I*_o_ that made no sense, while data point 23 was way off the rest of the points on the Benesi–Hildebrand plot, due to experimental errors associated with measurement of small differences in fluorescence intensity.

**Figure 3:**
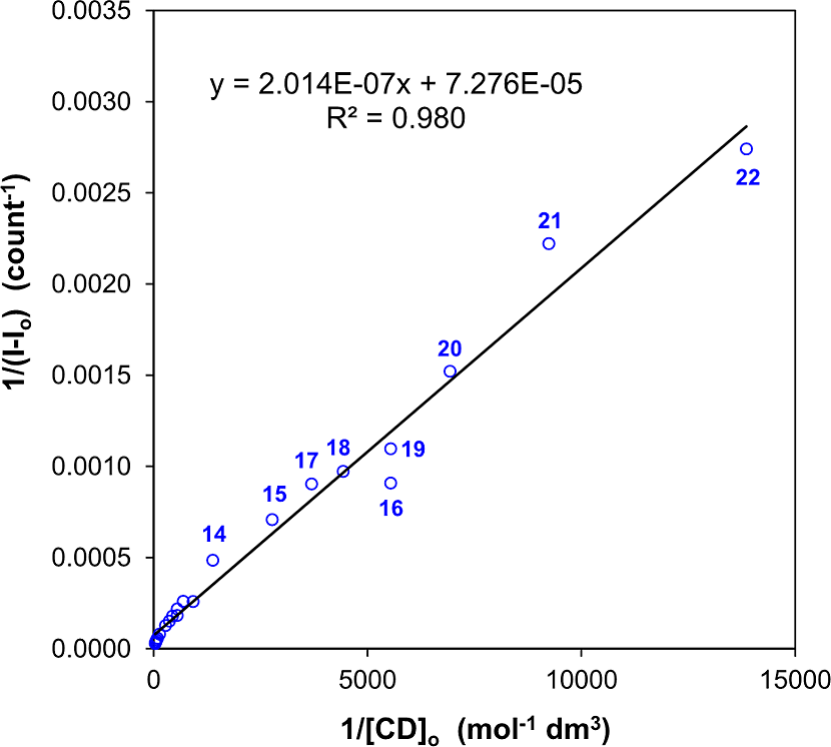
Example Benesi–Hildebrand plot for the data points 1–22 (Table 3), obtained for PAPC-CF_3_, (where: [CD]_o_—molar concentration of the cyclodextrin in solution; *I* and *I*_o_—the corresponding fluorescence intensities in the presence (*I*) and absence (*I*_o_) of cyclodextrin. The point numbers correspond to the data point numbers in Table 3).

A good linear relationship with a high correlation coefficient (*R* ^2^) between the reciprocal fluorescence intensity difference (1/(*I* – *I*_o_)) and the reciprocal cyclodextrin concentration (1/[CD]_o_) was obtained (Figure 3). This confirms that the fluorophore PAPC-CF_3_ forms a 1:1 inclusion complex with Captisol. However, when the host–guest association constant was determined from that plot, it turned out to be 361 dm^3^ mol^–1^, while the maximum fluorescence intensity seemed to be 18,361 count. When these constants (denoted subsequently as *K*_BH_ and *I*_1BH_, respectively, to distinguish them from the *K* and *I*_1_ values determined by other methods) are substituted to the eq 3, derived by transformation of the modified Benesi–Hildebrand eq (eq 2), or to the exact relationship between fluorescence intensity and the cyclodextrin concentration (eq 12, reported in the next section), the calculated fluorescence intensities (*I*_calc‑BH_ and *I*_calc‑exact_ in Table 3) do not match the corresponding experimental intensities (*I*), as shown in Figure 4.

3 $$I_{calc‐BH}=\frac{I_{o}+I_{1}K{\left[CD\right]}_{o}}{1+K{\left[CD\right]}_{o}}$$

**Figure 4:**
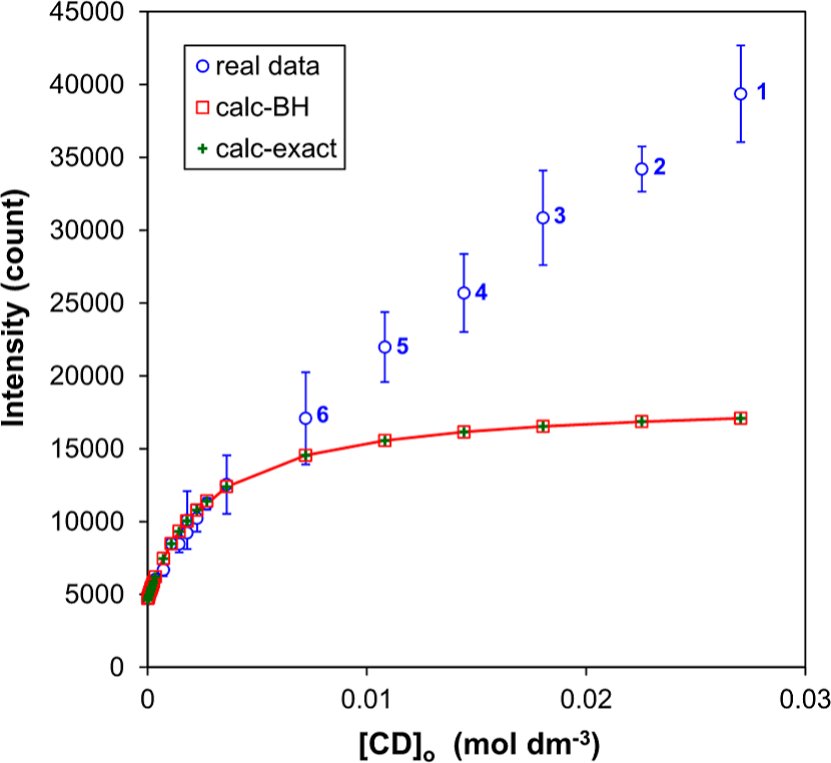
Mismatch between the intensities calculated on the basis of the *K*_BH_ and *I*_1BH_ values, determined from the Benesi–Hildebrand plot, and the real experimental data, when the top 22 data points from Table 3 were taken into account. The error bars correspond to standard deviations (σ_*n*–1_) of the average intensities, based on 3 independent replicates per each intensity measurement.

The fluorescence intensities calculated from the exact equation (eq 12) overlap with the intensities calculated from eq 3 (Figure 4). This indicates that in the case of PAPC-CF_3_, the assumption made during the derivation of the modified Benesi–Hildebrand equation (i.e., the assumption that the equilibrium concentration of cyclodextrin in solution [CD] was much higher than the concentration of the host–guest complex [FCD]) was always met within the entire range of the cyclodextrin concentrations studied. Hence, the problem is not with the approximate character of the Benesi–Hildebrand equation. The problem comes from incorrect *K*_BH_ and *I*_1BH_ values obtained by the Benesi–Hildebrand method, when all the data points are taken into account.

The difficulties with straightforward application of the Benesi–Hildebrand method to real experimental data come from the following factors:

1. Experimental data are never accurate and there are always some experimental errors associated with measured quantities, which affect the accuracy of the final results.
2 If a measured quantity (e.g., fluorescence intensity) is not a linear function of a varied parameter (e.g., cyclodextrin concentration), but the directly measured quantities are first transformed by some nonlinear mathematical functions to other variables to make the relationship linear (as in the case of the Benesi–Hildebrand equation, where the reciprocal of fluorescence intensity difference is treated as a linear function of reciprocal host concentration), then the errors associated with particular variables do not contribute to the same extent to the slope and intercept of the linear relationship. Then, selection of the most significant data points that least affect the final result accuracy is critical.
3 It has turned out that the linearity of the Benesi–Hildebrand relationship is not satisfactory enough to prove that the host–guest association constant, determined from its slope and intercept, matches well the experimental data because the data on the Benesi–Hildebrand plot (Figure 3) are not weighed evenly along the host concentration range. The data points corresponding to low CD concentrations contribute more to the slope, while being less accurate than the data at high concentrations. In order to verify visually the quality of match between raw experimental data and the data calculated theoretically on the basis of the determined constants, the raw original data (i.e., the measured fluorescence intensity and host concentrations) need to be compared directly on a common plot, such as that shown in Figure 4, not only on the linear Benesi–Hildebrand plot.

The incorrect *K*_BH_ and *I*_1BH_ values determined from the slope and intercept of the Benesi–Hildebrand plot (Figure 3) resulted from nonequal contribution of particular data points to the line slope and intercept, when the original data were reversed into reciprocals. When the reciprocals are calculated, small numbers give large reciprocals, while large numbers yield small reciprocals. So, the order of experimental data points in Figure 3 is reversed compared to that in Figure 4. Figure 3 demonstrates that in the Benesi–Hildebrand plot the data points corresponding to low cyclodextrin concentrations (i.e., the points 14–22) affected most the line parameters, while the remaining data points, accumulated near the plot origin, had little influence. Consequently, the association constant (*K*_BH_) and the maximum intensity (*I*_1BH_), determined by the Benesi–Hildebrand method, match well only the data points within low concentration range but do not necessarily fit to the entire concentration range, even when the Benesi–Hildebrand plot is linear. Moreover, at low cyclodextrin concentrations, the corresponding fluorescence intensity differences (*I* – *I*_o_) are smaller and harder to measure accurately than large intensity differences, corresponding to higher concentrations. Even small errors in the measurement of *I* and *I*_o_ may lead to large differences in 1/(*I* – *I*_o_) values, if the magnitudes of *I* and *I*_o_ are too close, which may have a deleterious effect on the accuracy of determination of the association constant (*K*). Therefore, if the fluorescence intensities, calculated from the constants determined from the Benesi–Hildebrand plot, are to match well the intensities measured experimentally, the intensity data (*I*), taken to the calculation, must not be too close to the initial intensity (*I*_o_).

Comparison of the raw experimental data with the data calculated theoretically on the basis of determined constants, such as that shown in Figure 4, is a good visual method that may be used for verification of whether the data range was selected correctly. For example, Figure 4 indicates that theoretically calculated fluorescence intensities corresponding to data points 1–6 do not coincide with experimental data points, so the *K*_BH_ and *I*_1BH_ values, determined from the Benesi–Hildebrand plot, using all the experimental data points, are simply wrong. Figure 4 also suggests that in the case of PAPC-CF_3_/Captisol complexation no data points below point 6 should be taken for determination of *K* and *I*_1_, if the theoretical relationship represented by eq 3 is to pass through those experimental data as close as possible.

When only data 1–6 (Table 3) are applied for determination of the *K* and *I*_1_ values, the Benesi–Hildebrand plot is also linear (Figure 5), but the line slope and intercept are different than those in Figure 3. Consequently, the association constant and the maximum intensity, determined on the basis of the limited number, but most significant 6 data points, (denoted subsequently as *K*_BH‑ltd_ and *I*_1BH‑ltd_) turned out to be *K*_BH‑ltd_ = 22.1 dm^3^ mol^–1^ and *I*_1BH‑ltd_ = 94,646 count. Visual verification of the correctness of the new *K* and *I*_1_ values, determined from Figure 5, is shown in Figure 6.

**Figure 5:**
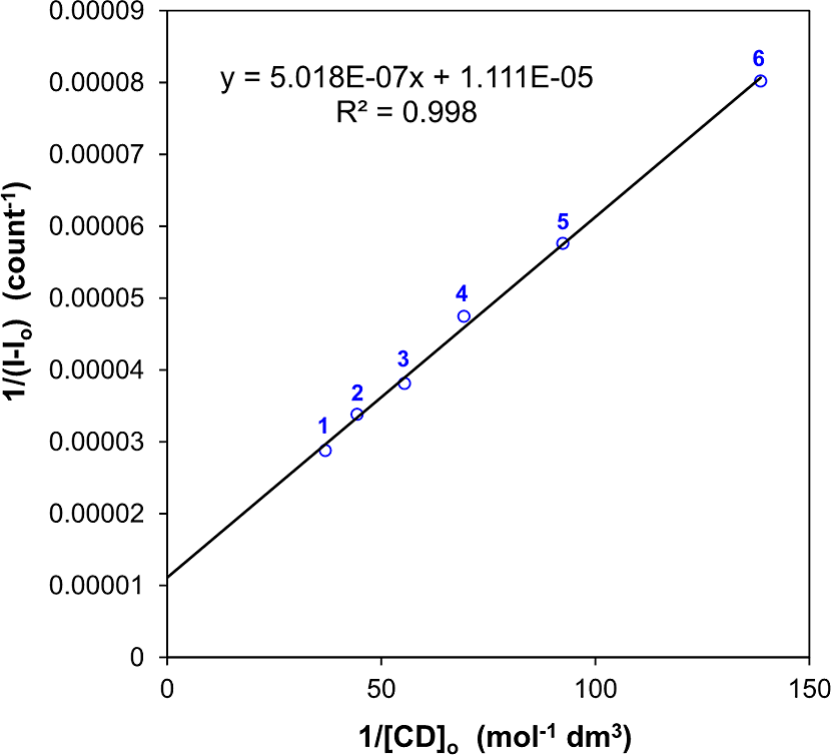
Benesi–Hildebrand plot for 6 most significant data, obtained for PAPC-CF_3_.

**Figure 6:**
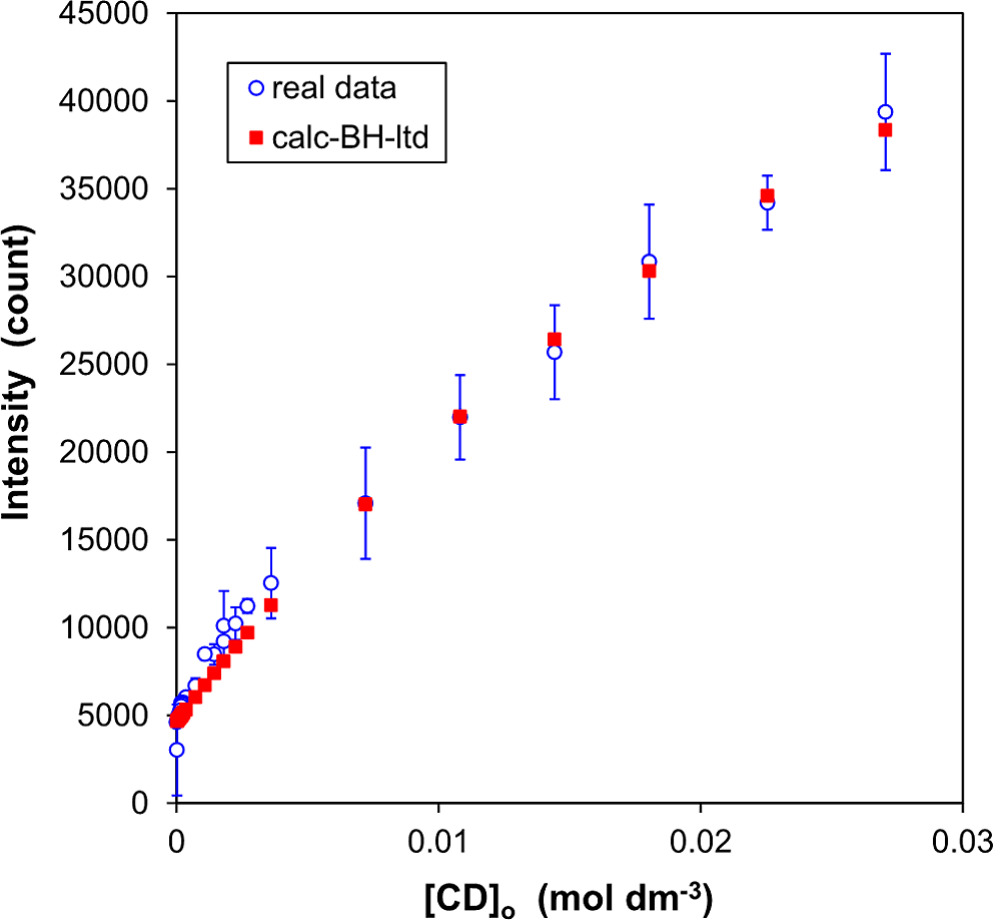
Comparison of the experimental data with the corresponding theoretical data, calculated on the basis of *K*_BH‑ltd_ and *I*_1BH‑ltd_ values, determined by the Benesi–Hildebrand method, using only 6 most significant data points.

Figure 6 indicates that by selection of only the most significant data for determination of *K* and *I*_1_ by the Benesi–Hildebrand method, a much better overlap between experimental data and the data calculated theoretically from the Benesi–Hildebrand equation can be achieved, better than in the case of use of all data that were not scattered in the Benesi Hildebrand plot. Hence, the association constant *K*_BH‑ltd_ = 22.1 dm^3^ mol^–1^ is much more reliable than the *K*_BH_. Then not only do the 6 experimental data points used for the determination of *K*_BH‑ltd_ and *I*_1BH‑ltd_ match well the calculated ones, but there is also a reasonably good match between all theoretical and experimental data points within the entire range of cyclodextrin concentrations studied (Figure 6). The optimal number of most significant data points for determination of *K* and *I*_1_ by the limited Benesi–Hildebrand method has to be found by a trial and error method, using visual verification (such as that shown in Figure 6), or other methods, until a satisfactory match between experimental and theoretical data is achieved within the entire range of host concentrations studied. The correlation coefficient (*R* ^2^) between experimental data and the data calculated from eq 3 for all experimental data points may be used as a quantitative indicator of match quality. We observed that within the cyclodextrin concentrations range studied, the top 6 data points (e.g., those for highest [CD]_o_) provided the best correlation coefficient (*R* ^2^) between experimental and calculated fluorescence intensities, so we applied the described data range selection method also to the experimental data obtained for the other pyridylcoumarins studied. The association constants (*K*_BH‑ltd_), determined by the Benesi–Hildebrand method, limited to the most significant data points, for all of the compounds studied, and their standard deviations from expected values are included in Table 4.

To date, not many authors reporting host–guest association constants (*K*), determined using the Benesi–Hildebrand method, were aware of the necessity of verification of the reliability of the determined constants by a direct comparison of raw experimental data with the data calculated on the basis of the found constants. Often linearity of the Benesi–Hildebrand plot was taken as satisfactory enough to assume that the determined association constants were correct, without any further verification. So, some of the host–guest association constants reported in literature may be off from their true physically meaningful values even by more than an order of magnitude, for the reasons demonstrated and explained in this paper.

#### 3.2.2 A Consecutive Iterations Method for Determination of Host–Guest Association Constants

In the 21st century, where PC computers capable of performing millions of calculations within seconds or even fractions of a second are available, linearization of equations in order to determine the characteristic constants from slopes and intercepts of straight lines matched to experimental data points is not necessary. Also, the assumptions required to make a complex equation linear are not necessary. The raw experimental data can be fitted directly to even very complex, nonlinear equations, while the characteristic constants may be determined by a consecutive iterations method, provided that an exact relationship between directly measured quantities is known.

The exact relationship between fluorescence intensity (*I*) and the cyclodextrin concentration [CD]_o_ for 1:1 host–guest systems can be easily derived by substitution of concentration balance equations at equilibrium (eqs 4 and 5) to the association constant definition (eq 1), where [*F*]_o_ and [CD]_o_ represent total molar concentrations of the reagents in measured solutions

4 $$\left[CD\right]={\left[CD\right]}_{o}-\left[FCD\right]$$

5 $$\left[F\right]={\left[F\right]}_{o}-\left[FCD\right]$$

6 $$K=\frac{\left[FCD\right]}{\left({\left[F\right]}_{o}-\left[FCD\right]\right)\left({\left[CD\right]}_{o}-\left[FCD\right]\right)}$$

Upon rearrangement of eq 6, a quadratic equation relative to the equilibrium concentration of the fluorophore-cyclodextrin complex [FCD] is obtained (eq 7)­

$${\left[FCD\right]}^{2}-\left[FCD\right]\left({\left[F\right]}_{o}+{\left[CD\right]}_{o}+\frac{1}{K}\right)+{\left[F\right]}_{o}{\left[CD\right]}_{o}=0$$ 7

From eq 7 the equilibrium concentration of host–guest complex [FCD] can be derived. The quadratic equation has two roots. A physically meaningful root, which ensures that the concentration of complex [FCD] does not exceed that of the limiting reagent [*F*]_o_, is given by eq 8.

$$\left[FCD\right]=\frac{1}{2}\left({\left[F\right]}_{o}+{\left[CD\right]}_{o}+\frac{1}{K}\right)-\frac{1}{2}\cdot \sqrt{{\left({\left[F\right]}_{o}+{\left[CD\right]}_{o}+\frac{1}{K}\right)}^{2}-4{\left[F\right]}_{o}{\left[CD\right]}_{o}}$$ 8

Eq 8 provides the equilibrium concentration of the inclusion complex ([FCD]) as a function of the initial concentrations of the reactants and the association constant (*K*). Analogous equation was reported previously by Thordarson, but unfortunately in his original paper, that equation was printed with errors, which were corrected later on.^44^ So, we decided to derive it again to make sure that the readers of this paper will know its origin.

The fluorescence intensity emitted by a solution containing both a fluorophore and a cyclodextrin is the sum of the intensities emitted by free fluorophore molecules (*F*) and that emitted by the fluorophore–cyclodextrin complex (FCD) at equilibrium. Hence, the measured intensity (*I*) is proportional to the concentrations of both emissive components (eq 9), where *k*_1_ and *k*_2_ are proportionality coefficients dependent on fluorescence efficiencies of the free and the complexed fluorophore molecules, respectively

9 $$I=k_{1}\left[F\right]+k_{2}\left[FCD\right]$$

It can be easily noticed that in the absence of cyclodextrin, where [FCD] = 0, the fluorescence comes only from the free fluorophore and its intensity (*I*_o_) is proportional only to the total fluorophore concentration [*F*]_o_ (eq 10). On the other hand, in the presence of a large excess of cyclodextrin, large enough to bind almost all of the fluorophore molecules, so that [*F*] ≈ 0 and [FCD] ≈ [*F*]_o_, the fluorescence intensity would reach its maximum value (*I*_1_) (eq 11).

10 $$I_{o}=k_{1}{\left[F\right]}_{o}$$

11 $$I_{1}=k_{2}{\left[F\right]}_{o}$$

Now, when the proportionality coefficients *k*_1_ and *k*_2_ are derived from eqs 10 and 11 and substituted to eq 9, followed by substitution of the equilibrium fluorophore concentration [*F*], represented by eq 5, and finally, substitution of [FCD] concentration expressed by eq 8, the exact relationship between the fluorescence intensity (*I*) and the cyclodextrin concentration ([CD]_o_) is obtained after rearrangement (eq 12), where *K* and *I*_1_ are the constants to be found by fitting eq 12 to experimental data.

$$I=I_{o}+\frac{I_{1}-I_{o}}{2}\left(1+\frac{{\left[CD\right]}_{o}}{{\left[F\right]}_{o}}+\frac{1}{K{\left[F\right]}_{o}}\right)\cdot [1-\sqrt{1-\frac{4{\left[F\right]}_{o}{\left[CD\right]}_{o}}{{\left({\left[F\right]}_{o}+{\left[CD\right]}_{o}+\frac{1}{K}\right)}^{2}}}]$$ 12

Nowadays, many software packages, implemented in PC computers, provide algorithms for achieving the best match of experimental data points to any clearly defined function, by a consecutive iterations method, We applied the “Solver” function in the Excel spreadsheet for the determination of the *K* and *I*_1_ constants by a least-squares fit of all experimental data points (including *I*_o_) to eq 12.

An example arrangement of the data, obtained for PAPC-CF_3_, on an Excel spreadsheet and setting the Solver parameters before iterations is shown in Figure 7. The procedure involved the following steps:

1. Raw experimental data (i.e., *I* and [CD]_o_), obtained for each fluorophore/cyclodextrin system, such as the example data shown in the second and third column of Table 3, were copied into two adjacent columns on an Excel spreadsheet (e.g., columns A and B in Figure 7).
2. Formulas representing eq 12 were appropriately coded into a third column to calculate automatically the theoretical intensities (*I*_calc_) (e.g., to cells C6–C30 in Figure 7), corresponding to the particular cyclodextrin concentrations [CD]_o_, on the basis of initially assumed values of *K* and *I*_1_, placed in a pair of empty cells anywhere in the spreadsheet (e.g., in cells H2 and H3 in Figure 7). Initially, the value of *K* was assumed to be 1, but any other positive number could be used as well. On the other hand, the initial guess for *I*_1_ had to be greater than the maximal value of the intensity (*I*) within the data series taken into account to make sense because *I*_1_ represented the maximal fluorescence intensity that would be achieved if all of the fluorophore molecules were complexed with cyclodextrin. So, *I*_1_ = 100,000 count was assumed as the first guess because all of the intensities measured were below that.
3. Formulas calculating squares of deviations between the theoretical (*I*_calc_) and experimental (*I*) intensities (i.e., (*I*_calc_ – *I*)^2^) were placed in the next column (e.g., column D in Figure 7), and a formula representing the sum of the squared deviations (*s* ^2^) covering all the data points was entered in an empty cell (e.g., cell D2 in Figure 7). This sum was applied as a parameter to be minimized by consecutive iterations, by changing the values of *K* and *I*_1_.
4. Next, the Solver function, available in the “Data” menu of Excel was opened. The cell containing the sum of squared deviations was selected as the target for minimization, while the cells containing *K* and *I*_1_ were selected as the variables to be changed during iterations. The nonlinear GRG solving method was selected for the *s* ^2^ minimization. However, it has to be pointed out that in a freshly loaded Excel program, usually the Solver function is not available on the “Data” menu. It has to be loaded at its first use by selecting the “Solver” from the Excel Add-ins list on “File → Options → Add-ins” menu.
5. Finally, after clicking on the “Solve” button, the Solver found new values of *K* and *I*_1_ (by the consecutive iterations), which provided the best match between the intensities calculated theoretically (*I*_calc_) on the basis of eq 12 and the experimental intensities (*I*). This was achieved by minimization of the sum of squared deviations between experimental and theoretical fluorescence intensities (*s* ^2^).

**Figure 7:**
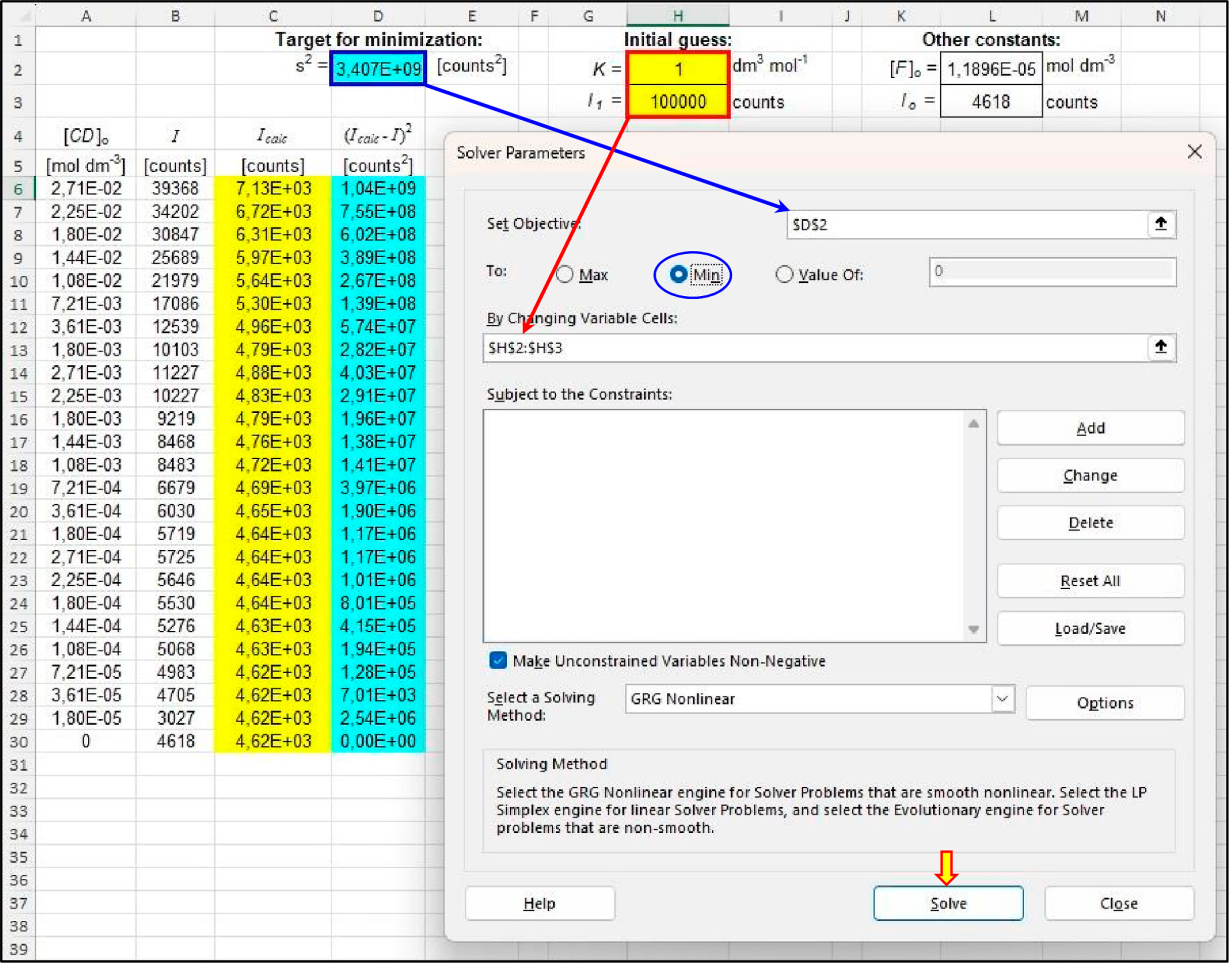
Example arrangement of the data on an Excel spreadsheet, used for the determination of *K* and *I*_1_ constants by the consecutive iterations method, before iterations.

Sometimes, when the experimental data points are too scattered, the consecutive iteration method using the Solver add-ins may not work. Hence, the data points affected by coarse errors that are way off from the general trend formed by the rest of the data have to be eliminated before applying the consecutive iterations method. Erroneous data can be easily identified by plotting the experimental values of the fluorescence intensity as a function of the cyclodextrin concentration. In the case of the PAPC compounds reported in this paper, the consecutive iterations method always worked well on all of the data points because there were no coarse errors.

Beside the visual verification, such as that shown in Figure 6, the quality of the match between the intensities, calculated on the basis of the *K* and *I*_1_ values, obtained using exact eq 12, and the experimental intensities (denoted subsequently as *K*_CI_ and *I*_1CI_, respectively), also can be verified visually by plotting the calculated intensities as a function of the measured ones, as shown in Figure 8 for PAPC-CF_3_ data. If there were no experimental errors in the intensity measurements, the data predicted using eq 12 would match exactly the intensities measured, and all of the data points in Figure 8 would lie on a straight line with a slope equal to 1. Figure 8 shows some data scattering on both sides of the straight line due to experimental errors, but what is the most important is that in the case of the consecutive iteration method, deviations between real intensities and the intensities calculated on the basis of exact eq 12 are minimized, unlike in the case of the Benesi–Hildebrand method, where deviations between reciprocals of the correlated quantities are minimized. Moreover, in addition to the visual verifications, the cross-correlation coefficient (*R* ^2^) between the experimental intensities and the predicted ones can be applied as a quantitative indicator of the reliability of determined association constants. The correlation coefficient, corresponding to the *K*_BH‑ltd_ and *I*_1BH‑ltd_ values, determined by the Benesi–Hildebrand method, limited to only 6 most significant data points (BH-ltd in Figure 8), is lower than the correlation coefficient corresponding to the *K*_CI_ and *I*_1CI_ values, determined by the consecutive iteration method, using all data points (CI in Figure 8). Hence, the association constant determined by the CI method can be considered as more accurate. In fact, it has been reported^45^ that many cyclodextrin association constants with various guest molecules, reported in literature, do not seem to be reliable, probably because of improper data workup procedures. In such cases the use of the consecutive iteration method (or other nonlinear fitting methods) may improve the results’ quality.

**Figure 8:**
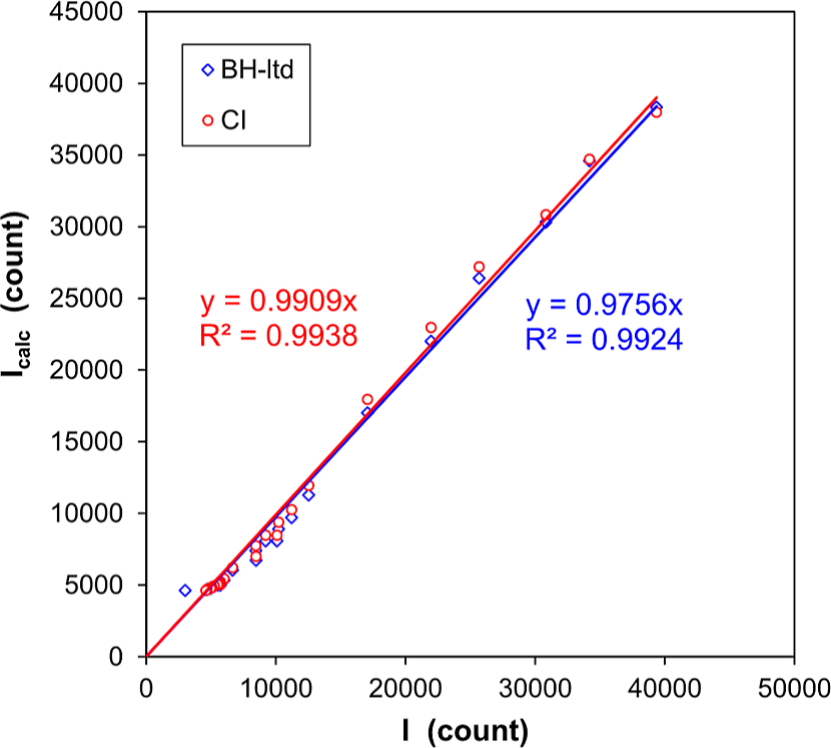
Correlation between the fluorescence intensities measured experimentally (*I*) and the intensities calculated theoretically (*I*_calc_) using eq 12, on the basis of *K*_BH‑ltd_ and *I*_1BH‑ltd_ values, determined by the limited Benesi–Hildebrand method (BH-ltd) or on the basis of *K*_CI_ and *I*_1CI_ values, determined by the consecutive iteration method (CI).

In summary, many physical or physicochemical constants are determined from slopes and intercepts of various linearized equations. The CI data workup methodology described in this paper is one of the nonlinear least-squares methods, called also nonlinear regressions. Mathematical principles of the nonlinear data fitting methods have been known for over a century. So, the CI methodology is not new. However, the advantage of the CI method, compared to other nonlinear data fitting methods, is that it can be easily implemented on any commercial spreadsheet program, such as Excel, without special skills in computer programming. Nevertheless, the other nonlinear fitting methods or global fitting models can be used as well, instead of less accurate methods based on linearized equations. What is important is that the fitting of experimental data to theoretical equations should be performed on directly measured variables, not on the secondary variables, obtained by transformation of original data using some nonlinear functions to make the theoretical relationship linear. Otherwise, the accuracy problems, demonstrated in his paper on the example of the use of the linear Benesi–Hildebrand equation, may arise. An excellent review on various data workup methodologies used in supramolecular chemistry for the determination of the host–guest association constant has been published by Thordarson,^44^ while a more general practical guide on curve fitting to experimental data points can be found in the book written by Motulsky and Christopoulos.^46^

#### 3.2.3 Stoichiometry of the Pirydylcoumarins/Captisol Complexes

It is well-known that cyclodextrins have good ability to form inclusion complexes with various organic molecules, sometimes at a stoichiometry other than 1:1.^47–49^ In particular, in the case of elongated molecules containing more than one binding site on opposite ends of the molecule, capable of interaction with polar groups within the cyclodextrin cavity, formation of 2:1 host/guest complexes is possible, where two cyclodextrin molecules bind to one guest molecule. Also some coumarin derivatives form 2:1 complexes.^50^ Structures of the PAPC compounds contain two basic nitrogen atoms capable of hydrogen bonding or other interactions, stronger than typical van der Waals forces. Hence theoretically, the pyridylcoumarins studied could also form 2:1 host–guest complexes with Captisol at high host concentrations. In order to verify that possibility, the Benesi–Hildebrand equation, corresponding to formation of the 2:1 host/guest complexes (eq 13), was attempted to fit to the experimental data corresponding to the highest range of Captisol concentrations, where formation of a 2:1 complex was most likely to appear (Figure 9)­

13 $$\frac{1}{I-I_{o}}=\frac{1}{\left(I_{1}-I_{o}\right)K_{2:1}}\cdot \frac{1}{{{\left[CD\right]}_{o}}^{2}}+\frac{1}{I_{1}-I_{o}}$$

where: *K*_2:1_ is the association constant of the 2:1 host/guest complex.

**Figure 9:**
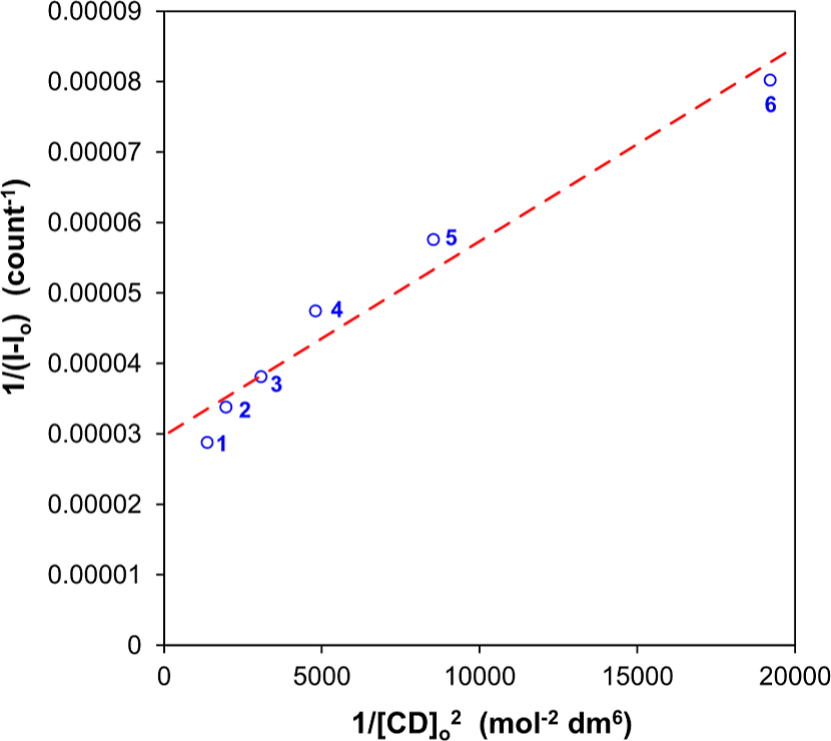
Example Benesi–Hildebrand plot for a 2:1 stoichiometry of the PAPC-CF_3_/Captisol complex.

Figure 9 indicates that the experimental data do not fit to the 2:1 binding model. The same data fit almost perfectly to the line corresponding to the 1:1 stoichiometry (Figure 5). This confirms that in aqueous solutions the pyridylcoumarins studied form only 1:1 inclusion complexes within the entire range of achievable Captisol concentrations.

On the other hand, the possibility of the formation of 1:2 inclusion complexes between Captisol and PAPCs can be ruled out on the basis of geometrical dimensions of the PAPC and Captisol structures because the β-CD cavity is simply too small to include (at least partially) more than one PAPC molecule.

### 3.3 Effect of Substituents on the Pyridylcoumarins/Captisol Host–Guest Association Constant

The association constants determined using the limited Benesi–Hildebrand method (*K*_BH‑ltd_) and those determined by the consecutive iteration method (*K*_CI_) for the pyridylcoumarin/cyclodextrin systems studied are collected in Table 4 and compared with the corresponding Hammett substituent constants (σ_p_).^51^ The association constants, determined by the limited Benesi–Hildebrand method, are slightly lower than those determined by the consecutive iterations method because in the case of the BH-ltd method only 6 most significant data points could be used to get a good match between theoretical and experimental data, for the reasons explained in this paper. Experimental errors associated with the measurement of small differences in fluorescence intensities (i.e., *I* – *I*_o_) at low cyclodextrin concentrations made it impossible to get a reasonable match between the experimental data and the data calculated on the basis of eq 3 or eq 12, when more than 6 data points, corresponding to highest cyclodextrin concentrations, were applied. On the other hand, all of the experimental data points could be used for the *K*_CI_ determination by the consecutive iterations method because the errors associated with particular data points contributed to the same extent to the *K*_CI_ accuracy. Therefore, association constants *K*_CI_ can be considered as more accurate. Moreover, discarding a series of experimental data points that do not stand out of a general data trend (which is necessary in the case of use of Benesi–Hildebrand equation to get a reasonable match between theoretical and experimental data) is not a good scientific practice. In that sense the consecutive iterations method is better because it does not waste any experimental data.

Figure 10 shows the Hammett plot, which is the relationship between the logarithm of the association constants (*K*_X_) of substituted derivatives, normalized relative to the association constant of the nonsubstituted compound (*K*_H_) and the Hammett substituent constants (σ_p_). Both the *K*_BH_ and *K*_CI_ show the same decreasing trend with an increase of σ_p_. This indicates that electron-withdrawing substituents, such as CF_3_ or CN, located in the para-position of the phenylamino moiety, decrease the association constant (*K*) of the 7-phenylamino-3-(2-pyridyl)­coumarins with Captisol, while electron-donating substituents exert an opposite influence. The magnitude of the slope, corresponding to the Hammett ρ-value for the complexation of the pyridylcoumarins with the cyclodextrin, which is of the order of −0.6 to −0.8, is relatively large. This suggests that interaction of the phenylamino moiety, which is most affected by the substituents, with the cyclodextrin dominates. Moreover, the negative ρ-value indicates that the interaction is stronger when the electron density within the phenylamino moiety or on the NH group is higher. This means that the pyridylcoumarins interact with positively charged sites within the Captisol cavity, possibly via hydrogen bonds.

**Figure 10:**
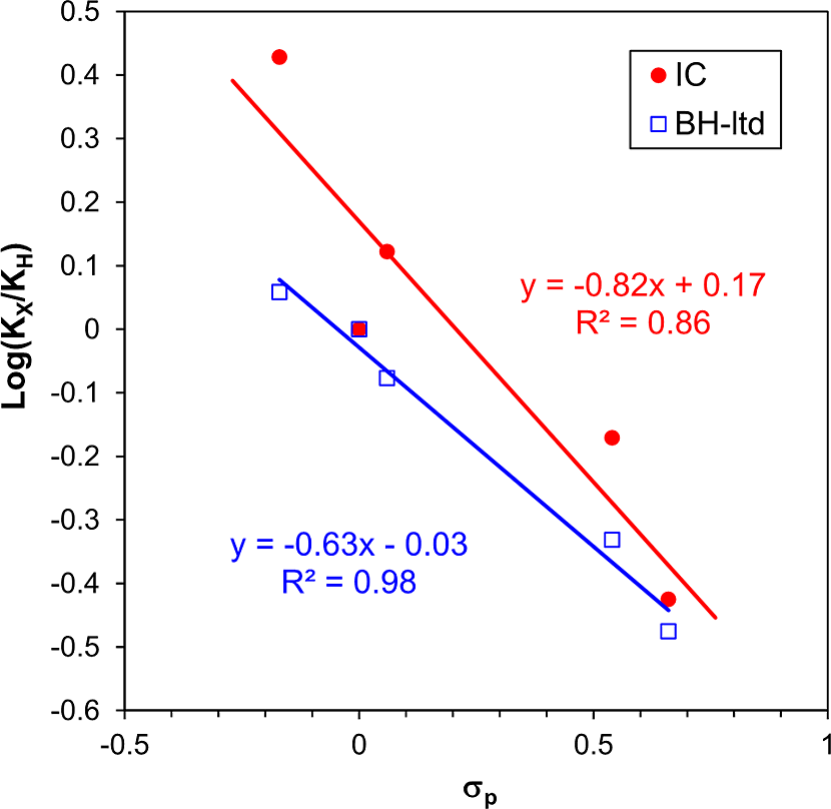
Hammett plot showing the effect of substituents on the association constant (*K*) of the pyridylcoumarins with Captisol (where: *K*_X_ – association constant of the pyridylcoumarin substituted with substituents X; *K*_H_ – the association constant of unsubstituted pyridylcoumarin; σ_p_ – Hammett constant of the substituent).

## 4 Conclusions

All of the 7-phenylamino-3-(2-pyridyl)­coumarins’ fluoresce, when excited within their absorption range. From among the derivatives studied, PAPC-CF_3_ and PAPC-CN show the highest fluorescence intensity that makes them the best choice for their practical application as fluorescent probes or stains. The fluorescence efficiency of the other pyridylcoumarins in an acetonitrile solution is more than an order of magnitude lower than that of PAPC-CF_3_.

The 7-phenylamino-3-(2-pyridyl)­coumarins form host–guest inclusion complexes with sulfobutylated-β-cyclodextrin (Captisol). Incorporation of the pyridylcoumarins into the cyclodextrin cavity increases their fluorescence intensity, which allows the determination of their host–guest association constants by fluorescence spectroscopy. The association constant of the pyridylcoumarins with Captisol varies within the range of 17–122 dm^3^ mol^–1^, depending on the substituent.

Application of the Benesi–Hildebrand method for determination of a host–guest association constant may lead to incorrect results, if the range of host concentrations covers more than 1 order of magnitude, because the data points corresponding to low host concentrations, which are least accurate, contribute most to the slope and intercept of the Benesi–Hildebrand plot, while the data corresponding to high host concentrations, which are most significant, exert negligible effect on the line parameters. Therefore, linearity of the Benesi–Hildebrand plot alone cannot be taken as satisfactory enough evidence that the host–guest association constant, determined from the Benesi–Hildebrand plot, is correct. Verification of the correctness of determined constants is always necessary by a direct comparison of raw experimental data to those calculated on the basis of the found constants.

Reasonably accurate host–guest association constants still may be obtained by the Benesi–Hildebrand method when only the most significant data points are taken to the Benesi–Hildebrand plot, using the data selection methodology described in this paper. However, the most reliable host–guest association constants can be determined by fitting all of the nonscattered experimental data points to the accurate equation (eq 12), by the nonlinear least-squares method based on consecutive iterations.

The strength of the interaction of 7-phenylamino-3-(2-pyridyl)­coumarins with Captisol decreases with an increase of the electron-withdrawing character of the substituent in the para-position of the phenylamino moiety, which indicates that the pyridylcoumarins studied interact with positively charged sites within the cyclodextrin cavity.

In general, linearization of various nonlinear relationships for the purpose of determination of physical or physicochemical constants from slopes and intercepts of the linear equations is very common. The data presented in this paper demonstrate how misleading such linearizations can be, if the accuracy of the constants determined from linearized relationships is not verified by a comparison of directly measured (i.e., not transformed) quantities with the data calculated on the basis of the original nonlinear relationship. Therefore, in the cases where the relationship between directly measured quantity and the corresponding independent variable (or variables) is nonlinear, we recommend using nonlinear fitting methods, such as the CI method described in this paper. Perfect fit of experimental data to a linearized relationship proves only that the original nonlinear equation describes well the relationship studied, while a nonlinear fitting method should be used for precise determination of characteristic constants.

## Supplementary Material



## Notes

^1^H NMR spectra of the compounds studied, the corresponding ^13^C NMR spectra and experimental data obtained for all of the compounds studied. These data may be utilized by readers of this paper for practicing the data workup methodology, described in Section 3.2.2, before its application to their own experimental data (PDF)
