# Supplementary material for: New Fluorescent Probes, Their Spectroscopic Properties, and an Iterative Analysis of Their Complexation with Cyclodextrins
Source: J Phys Chem B. 2026 Feb 3;130(6):1849–64. doi: 10.1021/acs.jpcb.5c06255 (PMC13296734; doi:10.1021/acs.jpcb.5c06255)
Supplement: Supplementary file 1 [file jp5c06255_si_001.pdf]

New Fluorescent Probes, their Spectroscopic Properties and Iterative Analysis of their Complexation with Cyclodextrins

Monika Topa-Skwarczyńska [a], Patryk Szymaszek [a], Anna Chachaj-Brekiesz [b], Mariusz Galek [c], Joanna Ortyl [a,c,d], Roman Popielarz [a,]*

[a] *Cracow University of Technology, Faculty of Chemical Engineering and Technology, Warszawska 24, 31-155 Kraków, Poland*

[b] *Jagiellonian University, Faculty of Chemistry, Gronostajowa 2, 30–387 Kraków, Poland*

[c] *Photo HiTech Ltd., Bobrzyńskiego 14, 30-348 Kraków, Poland*

[d] *Photo4Chem Ltd., Lea 114, 30-133 Kraków, Poland*

* Corresponding author: Roman Popielarz
e-mail: roman.popielarz@pk.edu.pl

^{1}H NMR Spectra of the Compounds Studied

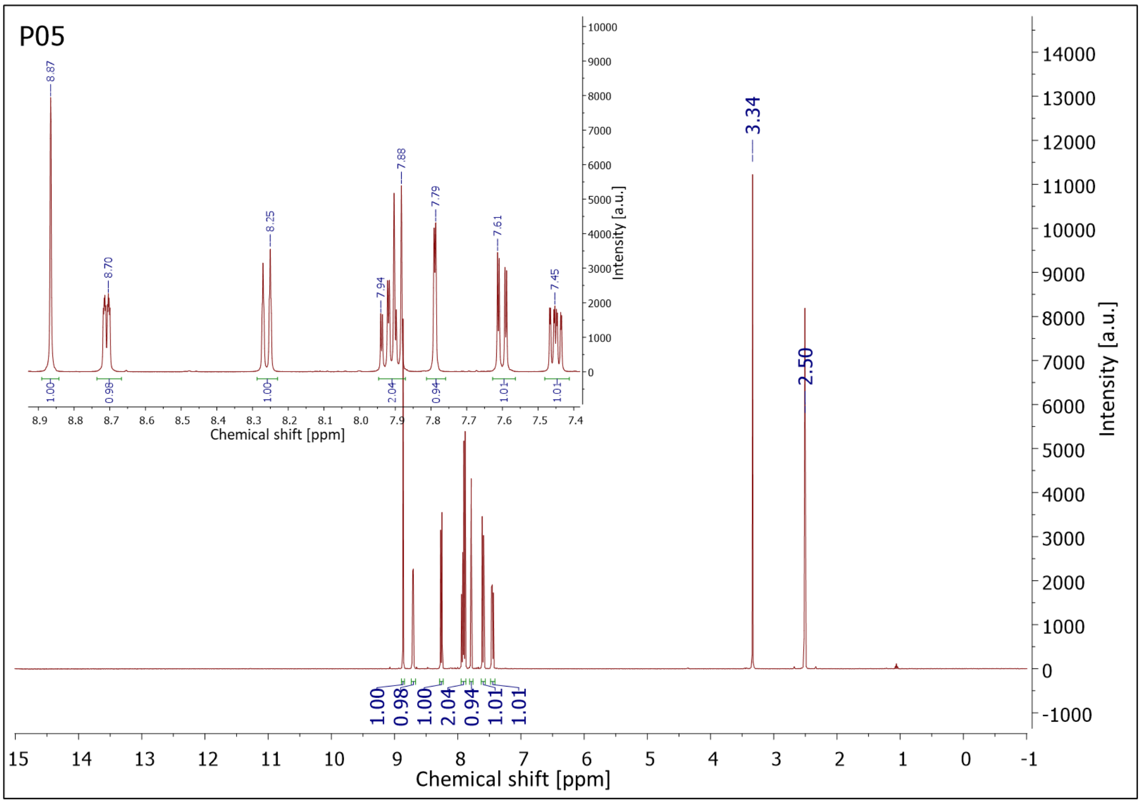

Figure S1. ^{1}H NMR spectrum of 7-bromo-3-(2-pirydyl)coumarin (an intermediate).

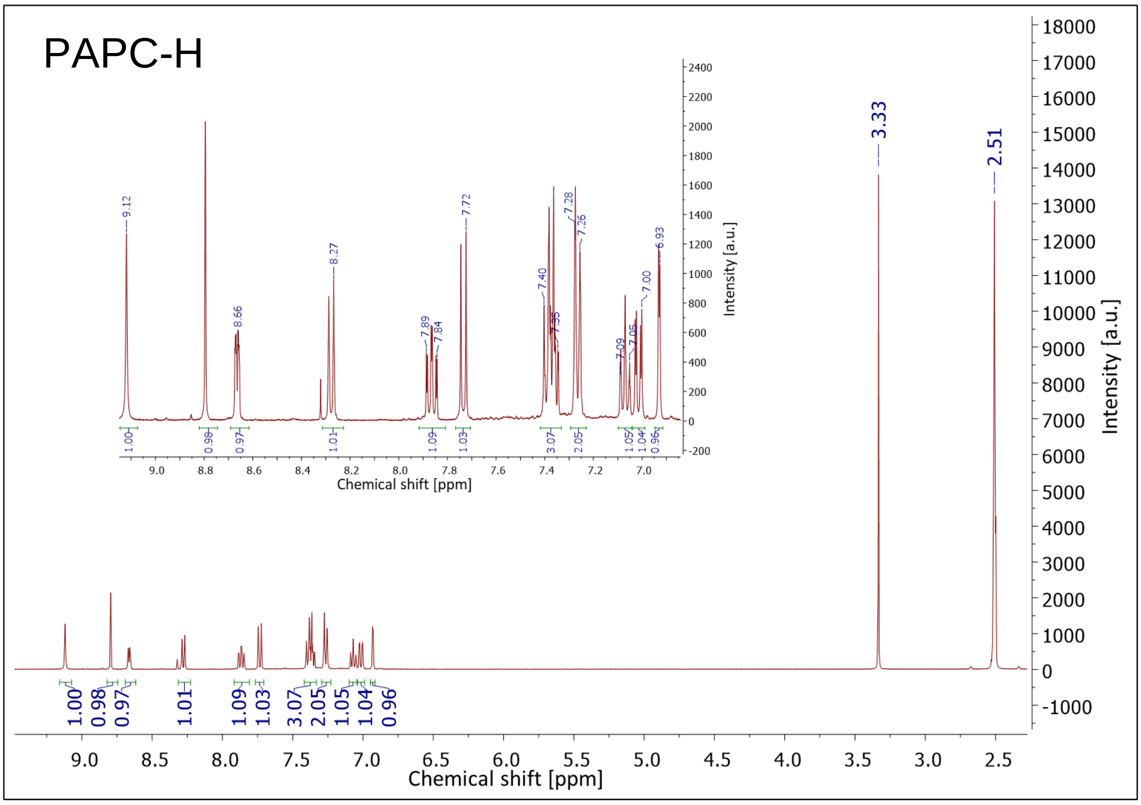

Figure S2. ^{1}H NMR spectrum of 7-phenylamino-3-(2-pyridyl)coumarin (PAPC-H).

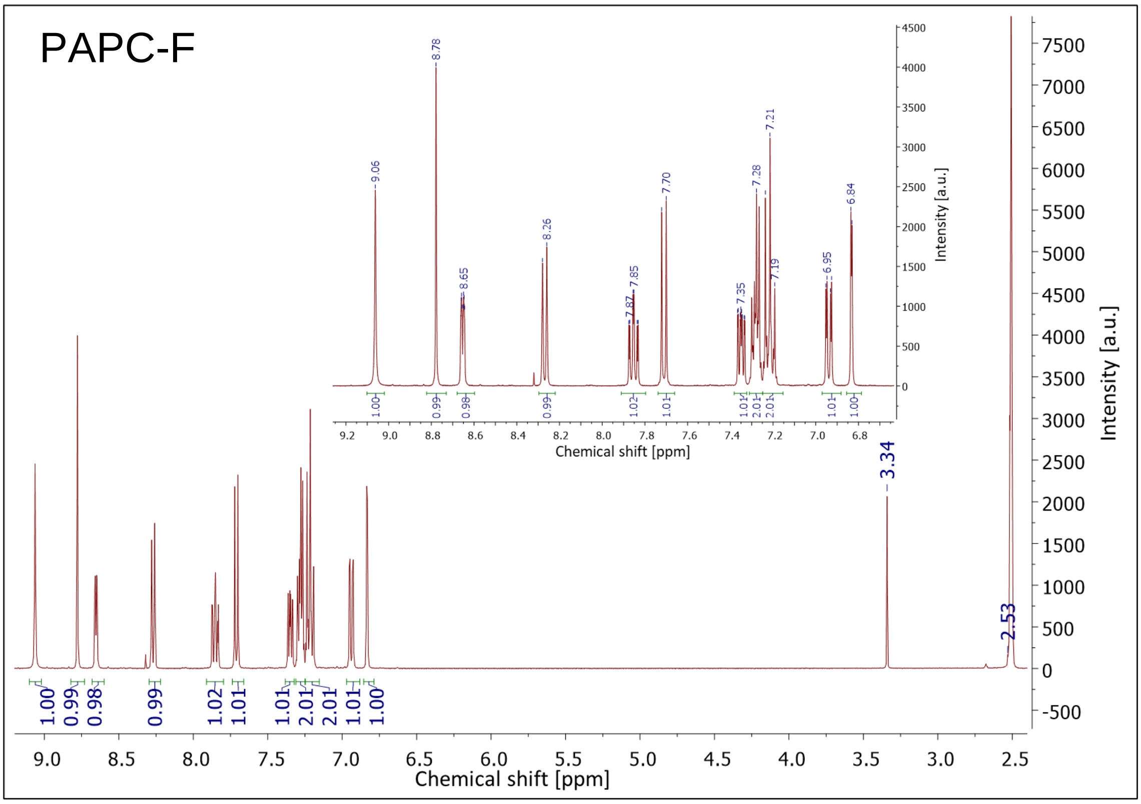

Figure S3. ^{1}H NMR spectrum of 7-(4-fluorophenyl)amino-3-(2-pyridyl)coumarin (PAPC-F).

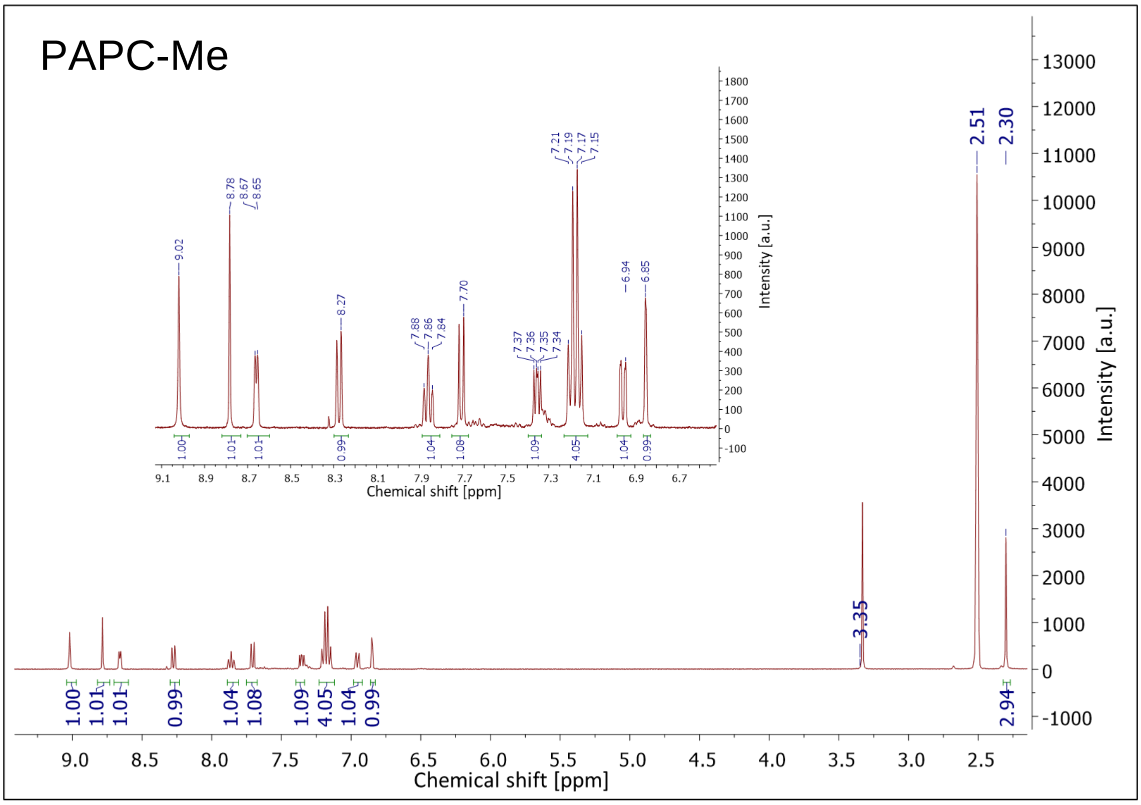

Figure S4. ^{1}H NMR spectrum of 7-(4-methylphenyl)amino-3-(2-pyridyl)coumarin (PAPC-Me).

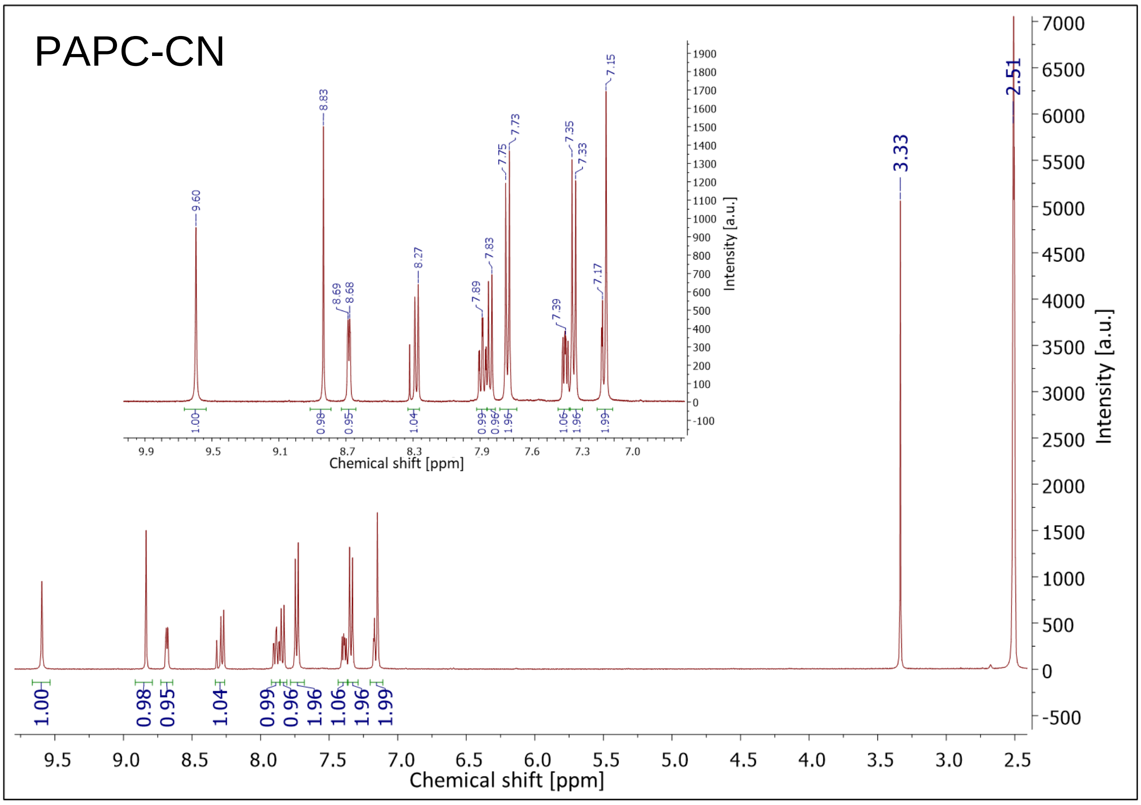

Figure S5. ^{1}H NMR spectrum of 7-(4-cyanophenyl)amino-3-(2-pyridyl)coumarin (PAPC-CN).

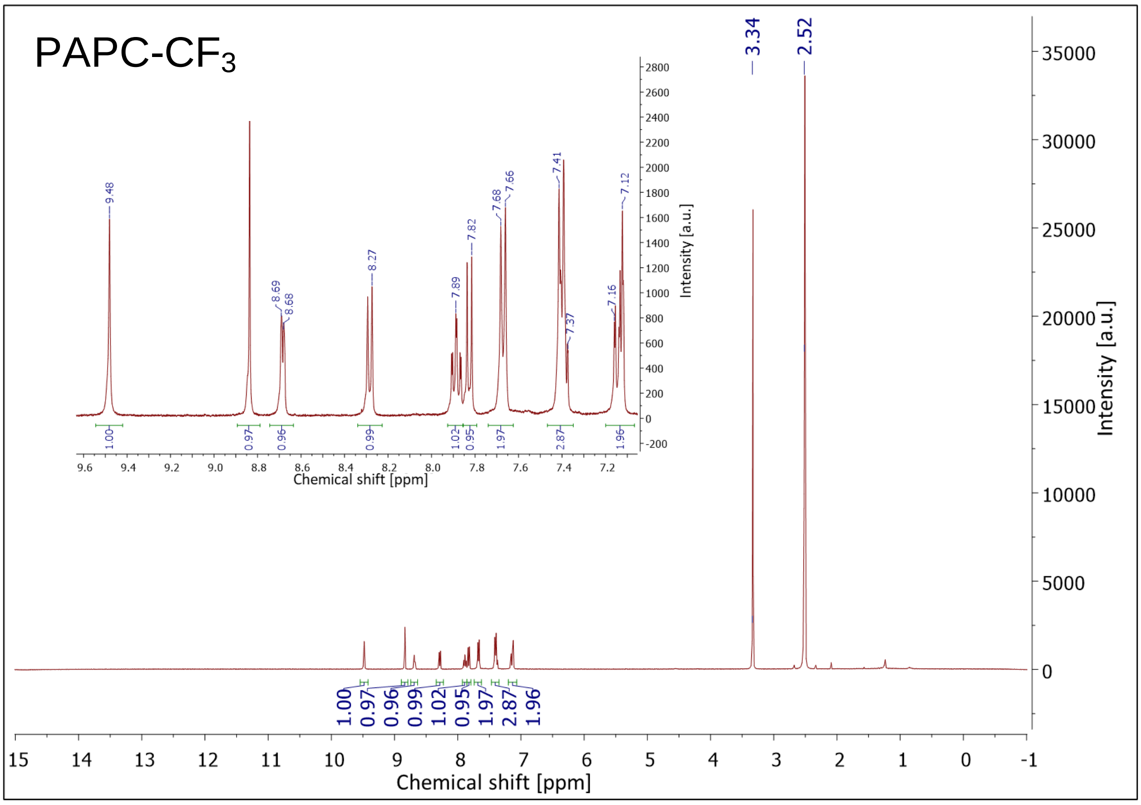

Figure S6. ^{1}H NMR spectrum of 7-(4-trifluoromethylphenyl)amino-3-(2-pyridyl)ccoumarin (PAPC-CF_3).

^{13}C NMR Spectra of the Compounds Studied

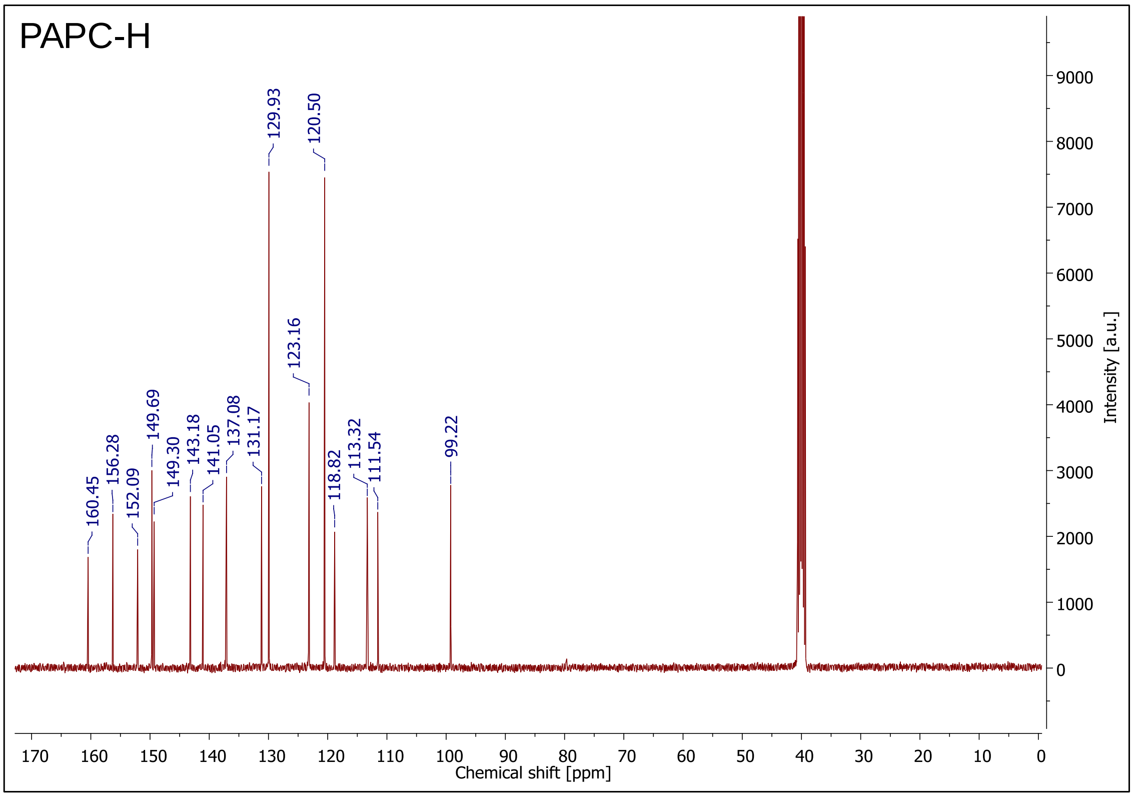

Figure S7. ^{13}C NMR spectrum of 7-phenylamino-3-(2-pyridyl)coumarin (PAPC-H).

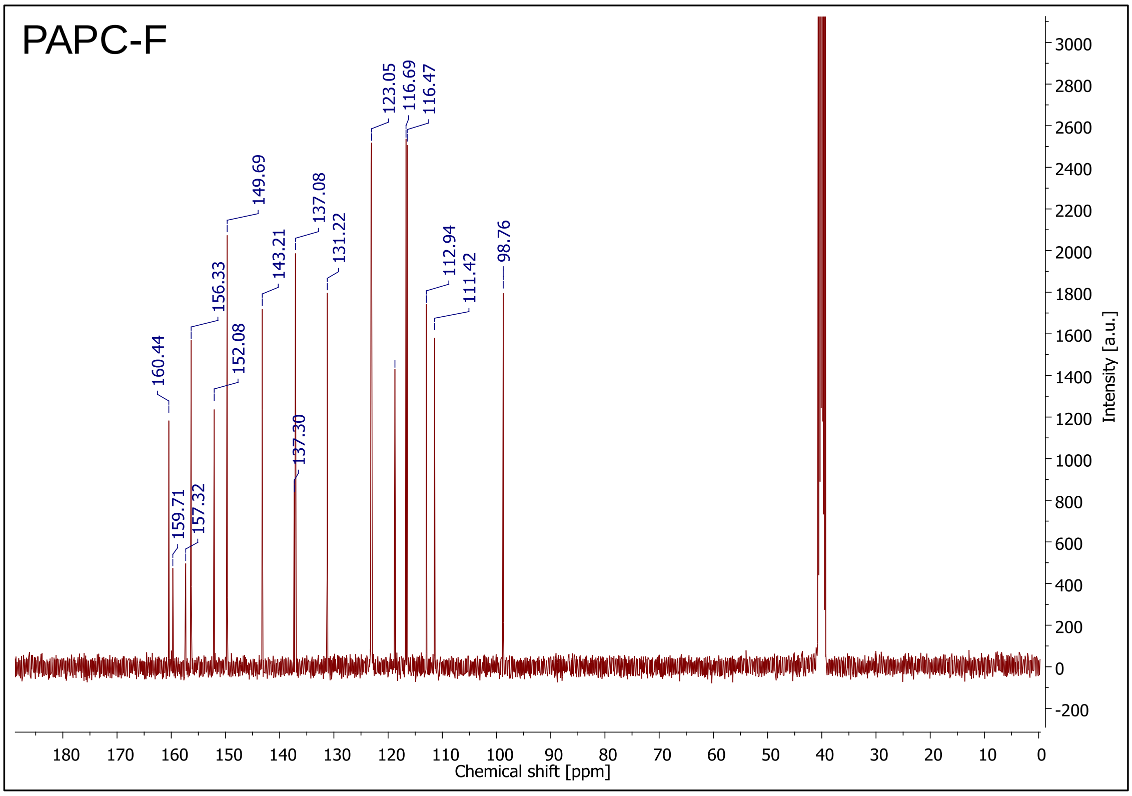

Figure S8. ^{13}C NMR spectrum of 7-(4-fluorophenyl)amino-3-(2-pyridyl)coumarin (PAPC-F).

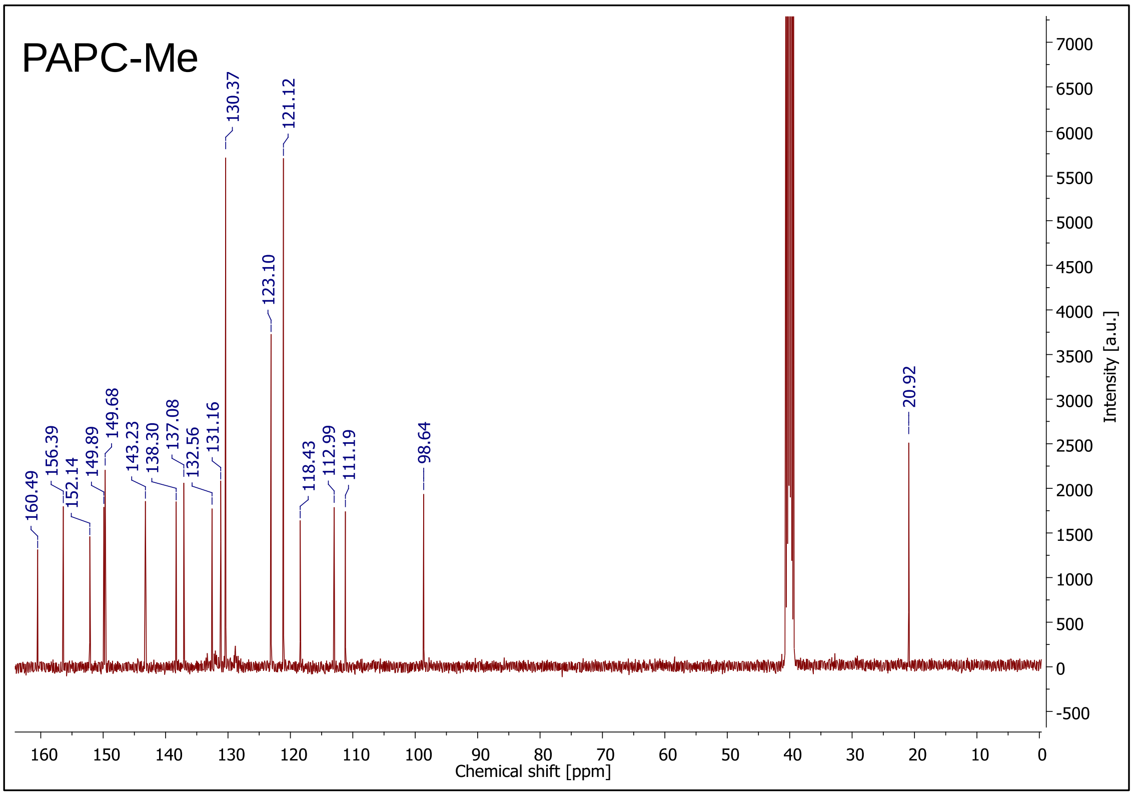

Figure S9. ^{13}C NMR spectrum of 7-(4-methylphenyl)amino-3-(2-pyridyl)coumarin (PAPC-Me).

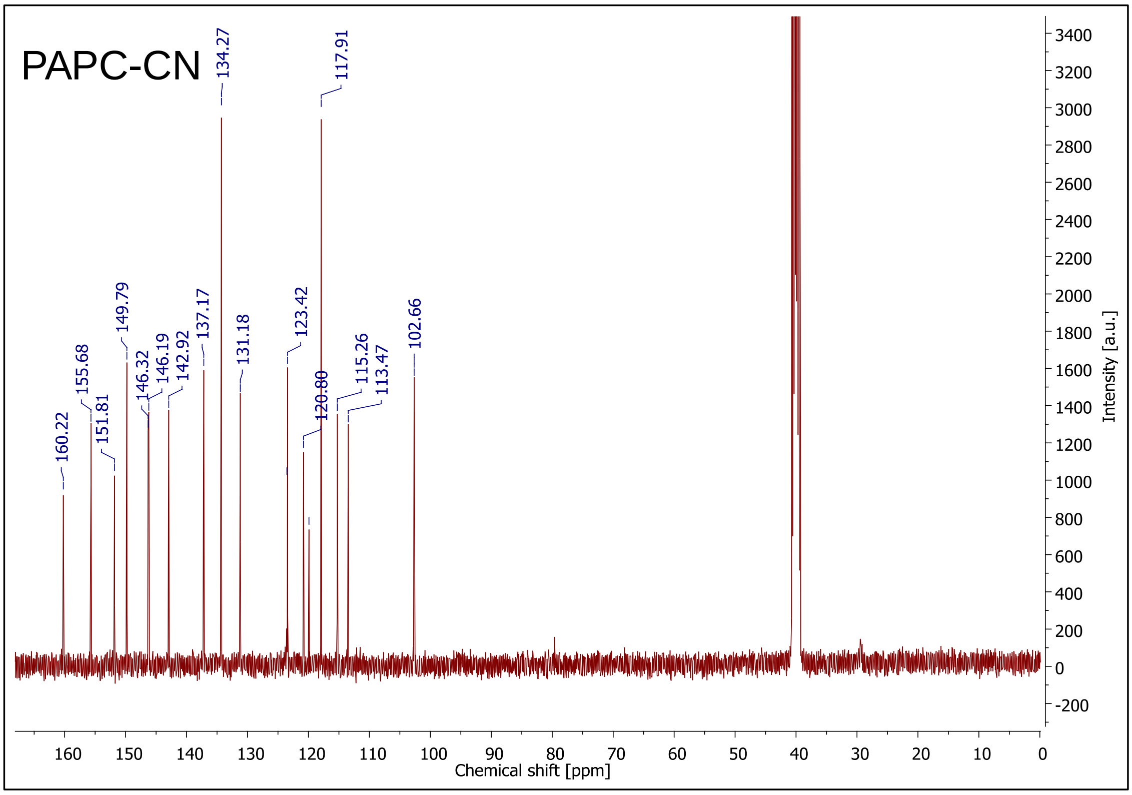

Figure S10. ^{13}C NMR spectrum of 7-(4-cyanophenyl)amino-3-(2-pyridyl)coumarin (PAPC-CN).

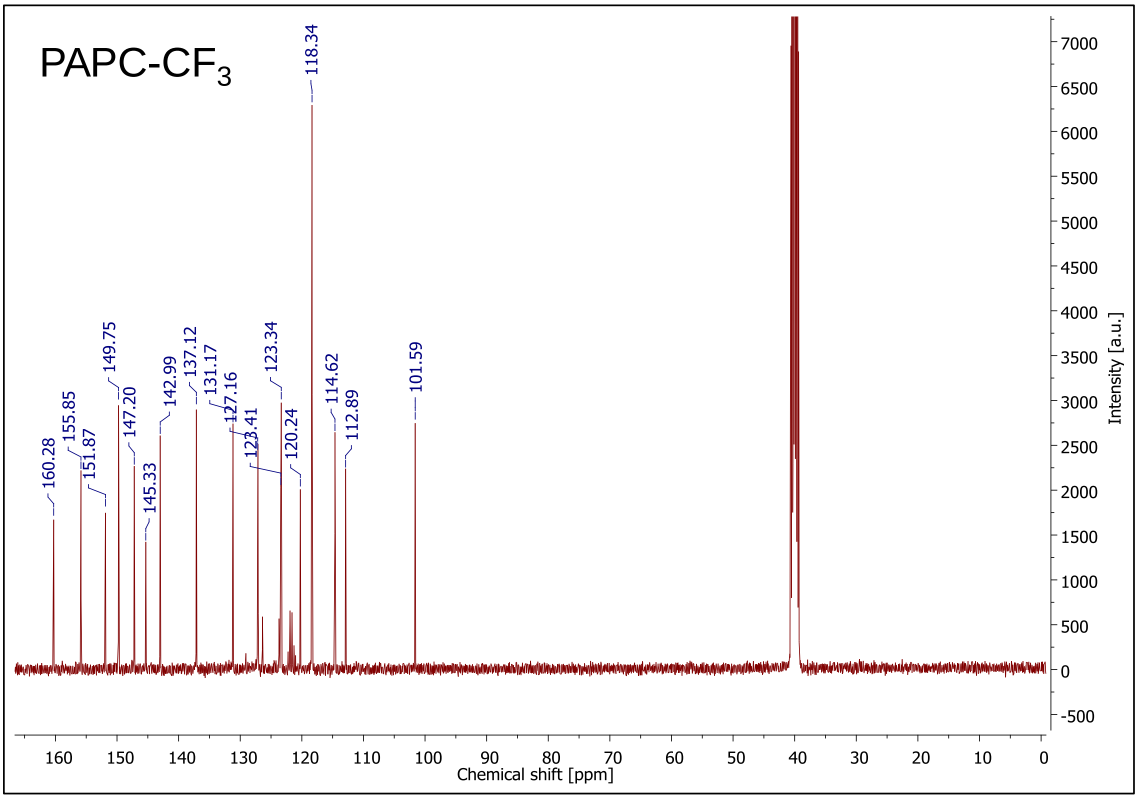

Figure S11. ^{13}C NMR spectrum of 7-(4-trifluoromethylphenyl)amino-3-(2-pyridyl)ccoumarin (PAPC-CF_3).

Experimental data obtained for complexation of the fluorophores studied with Captisol®

Table S1.
Average fluorescence intensities (I), obtained for constant concentration of the fluorophore ($[F]_o$) and various concentrations of Captisol® ($[CD]_o$).

$[F]_o$ [mol dm^{-3}] →	$1.447\cdot10^{-5}$	$1.385\cdot10^{-5}$	$1.369\cdot10^{-5}$	$1.190\cdot10^{-5}$	$1.340\cdot10^{-5}$
	***I* [counts]**				
$[CD]_o$ [mol dm^{-3}]	PAPC-H	PAPC-Me	PAPC-F	PAPC-CF3	PAPC-CN
$2.705\cdot10^{-2}$	43977.4	46125.2	42747.2	39368.0	42996.5
$2.255\cdot10^{-2}$	39012.8	41860.0	39734.5	34202.2	38572.9
$1.804\cdot10^{-2}$	34380.0	36895.2	33636.2	30847.1	32844.1
$1.443\cdot10^{-2}$	28888.0	33746.0	31276.2	25689.1	28259.1
$1.082\cdot10^{-2}$	25562.5	29241.3	26177.6	21979.3	23602.5
$7.215\cdot10^{-3}$	20348.2	26504.5	21079.0	17085.7	18544.6
$3.607\cdot10^{-3}$	13044.3	21020.4	16208.8	12539.2	12880.8
$1.804\cdot10^{-3}$	7904.1	19432.6	12388.3	10102.9	9699.2
$2.705\cdot10^{-3}$	10491.8	20940.8	13507.8	11227.0	11244.8
$2.255\cdot10^{-3}$	9926.6	19527.1	13018.7	10227.4	11097.8
$1.804\cdot10^{-3}$	8676.6	20304.0	11800.4	9219.5	10274.1
$1.443\cdot10^{-3}$	7010.9	18871.8	10860.2	8468.1	9553.2
$1.082\cdot10^{-3}$	5855.9	16640.4	9544.4	8482.6	8565.5
$7.215\cdot10^{-4}$	5090.8	15397.2	8727.7	6678.9	8150.1
$3.607\cdot10^{-4}$	3751.3	14093.8	7474.7	6030.0	7521.1
$1.804\cdot10^{-4}$	2949.8	12660.9	6597.6	5719.0	7109.3
$2.705\cdot10^{-4}$	3235.2	15776.6	6677.8	5725.2	6859.7
$2.255\cdot10^{-4}$	3167.8	13800.5	6603.2	5646.4	6941.6
$1.804\cdot10^{-4}$	3002.0	12707.2	7201.6	5529.8	6748.0
$1.443\cdot10^{-4}$	2824.2	12292.4	5839.7	5275.5	6196.3
$1.082\cdot10^{-4}$	2389.8	11502.2	5703.5	5068.2	6159.0
$7.215\cdot10^{-5}$	2522.8	11344.0	5667.1	4982.8	6397.8
$3.607\cdot10^{-5}$	2332.6	11259.9	5281.3	4705.1	6024.4
$1.804\cdot10^{-5}$	2111.2	10264.7	5039.8	3026.9	6182.0
0	2107.2	10240.2	5044.9	4617.9	5984.5
